# Unifying Composition and Process Design: A Heterogeneous Graph Neural Network for Discovering High‐Performance Cu Alloys

**DOI:** 10.1002/advs.202524364

**Published:** 2026-06-26

**Authors:** Jie Yin, Qian Lei, Wei Yan, Yue Li, Tong Xie, Shuang Zhou, Chong Wang, Bram Hoex, Qiang Long, Caoyang Jiang, Min Song, Zhou Li, Zhangwei Wang

**Affiliations:** ^1^ State Key Laboratory of Powder Metallurgy Central South University Changsha China; ^2^ State Key Laboratory of Advanced Fiber Materials, College of Materials Science and Engineering Donghua University Shanghai China; ^3^ Max Planck Institute For Sustainable Materials Düsseldorf Germany; ^4^ School of Photovoltaics and Renewable Energy Engineering University of New South Wales UNSW Sydney New South Wales Australia; ^5^ Green Dynamics Sydney New South Wales Australia; ^6^ The School of Automation Central South University Changsha China; ^7^ School of Materials Science and Engineering Central South University Changsha China

**Keywords:** composition and process design, heterogeneous graph neural network, high‐performance cu alloys, machine learning

## Abstract

The properties of copper alloys critical for sustainable development depend on their processing pathways. However, the complex and variable character of these pathways is incompatible with conventional machine learning models that require fixed‐input structures, creating a barrier to AI‐driven materials design. To address this challenge, we introduce a heterogeneous graph neural network to model the material system including both elemental composition and process steps as a unified graph. The graph integrates element and process nodes, interconnected by learnable edges representing the intricate relationships between composition and processing steps. This approach inherently accommodates variable‐length process pathways and bypasses manual feature engineering by directly utilizing native elemental properties. It effectively captures the complex coupling between composition and processing without suffering from data sparsity or dimensional explosion. Applying this framework, we designed and experimentally validated a novel copper alloy (Cu‐Cr‐Zr‐Y‐La‐Mg‐Zn) and tailored process, achieving an exceptional combination of 710 MPa in yield strength, 726 MPa in tensile strength, and 75% IACS (International Annealed Copper Standard) in electrical conductivity. This work presents a new approach for materials discovery, offering a scalable solution to co‐optimize composition and processing for materials defined by complex manufacturing histories.

## Graphical abstract

By overcoming the fixed‐path limitations of conventional machine learning, a heterogeneous graph neural network fundamentally reconstructs material data representation. Integrating variable processing sequences with intrinsic elemental features, this framework enables exploratory optimization across high‐dimensional spaces. This approach successfully discovers a novel copper alloy exhibiting an exceptional, previously inaccessible synergy of ultrahigh strength and electrical conductivity.

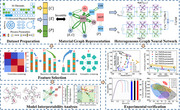

## 1 Introduction

Driven by the global consensus on sustainable development, breakthroughs in high‐strength, high‐conductivity copper alloys are critical for the green transformation of multiple sectors [1, 2, 3]. As core materials for electrification infrastructure, their performance directly impacts environmental sustainability. Higher strength facilitates structural lightweighting, thereby reducing energy consumption in transportation; meanwhile, higher conductivity diminishes energy losses during power transmission and conversion. Consequently, developing copper alloys that combine these properties provides fundamental support for deep decarbonization in energy‐intensive fields such as clean energy and electric transportation [2, 3]. However, an inherent trade‐off exists between strength and conductivity in copper alloys. This trade‐off is significantly influenced by thermomechanical treatment (TMT) and composition, creating a complex multi‐objective optimization problem [3, 4].

At this pivotal moment, artificial intelligence (AI) offers unprecedented possibilities for achieving these goals, which drives a profound transformation in materials science research paradigms [5, 6, 7, 8, 9, 10, 11]. However, a significant frontier challenge remains in applying these advanced computational techniques to the optimization of TMT, which integrates sequences of plastic deformation (e.g., rolling, forging) and thermal treatment (e.g., annealing, quenching) [3, 12, 13]. Conventional machine learning (ML) models, constrained by requirements for fixed‐structure inputs, struggle to effectively handle the inherent heterogeneity and variability intrinsic to TMT design. This variability manifests in two critical dimensions: the topological variability stemming from the dynamic inclusion or exclusion of specific process modules, and the temporal variability associated with the reconfigurable sequencing of these modules. The resulting sparse, high‐dimensional input space, arising from diverse process pathways, introduces a severe curse of dimensionality dilemma. This core limitation confines existing AI‐driven optimization to rigid, pre‐defined process chains, thereby preventing the effective navigation and exploration of the dynamic and expansive search space of all possible TMT pathways [14, 15, 16, 17].

The challenge of heterogeneous process variability critically constrains not only process optimization but also the concurrent co‐design of alloy compositions. Because conventional ML models demand a fixed, uniform input structure for the process path, a substantial amount of valuable data from distinct processes must often be discarded, retaining only narrow subsets for training [2, 18]. This self‐imposed data scarcity is a critical bottleneck, as limited samples frequently lead to model underfitting or overfitting, thereby undermining the potential of ML for generalized materials design. Although approaches like ensemble learning [19, 20, 21], transfer learning [22], active learning [17, 23], and feature engineering [24] aim to address this, these methods often prove insufficient. Transfer learning, for example, often fails without massive, relevant source datasets [22], and feature engineering is limited to combining simple elemental features rather than leveraging intrinsic material properties directly [2, 18, 24], which would reintroduce high‐dimensional sparsity.

To address these limitations, graph neural networks (GNNs) offer a promising alternative paradigm with inherent architectural advantages for handling heterogeneous and variable‐length data. Within the broader ML community, heterogeneous graph structures are well recognized for their ability to uniformly represent diverse node types (e.g., element nodes and process nodes) alongside their complex relational dependencies. This approach fundamentally circumvents the information distortion and curse of dimensionality caused by the forced padding of variable‐length sequences. Furthermore, the message‐passing mechanism in GNNs enables adaptive information aggregation without being constrained by data scale, order, or topological configuration, while directly leveraging intrinsic elemental attributes to eliminate reliance on hand‐crafted feature engineering. Building on these advantages, GNNs have achieved significant success across frontier domains, such as graph‐level molecular property prediction [6] and accelerated DFT calculations [5]. However, the tailored application of GNNs to process‐aware alloy design, particularly for synergistically optimizing composition and path‐dependent thermomechanical histories, remains largely unexplored.

In this work, we introduce a heterogeneous graph neural network (HGNN) that transforms the material system into a unified graph of element and process nodes with learnable edges. The architecture learns directly from intrinsic elemental features and captures the interplay between composition and processing through dynamic node interactions. We apply this framework to the multi‐objective design of high‐performance Cu alloys [3, 4]. Guided by the model, we designed a novel multi‐element composition (Cu‐Cr‐Zr‐Y‐La‐Mg‐Zn) and a corresponding multi‐stage TMT process. The alloy was experimentally validated to achieve a tensile strength of 726 MPa and an electrical conductivity of 75% IACS. This demonstrates that our graph‐based approach offers a useful and interpretable solution for complex materials design problems involving intricate processing pathways.

## 2 Results and Discussions

### 2.1 Heterogeneous Graph Learning Model Workflow

**FIGURE 1:**
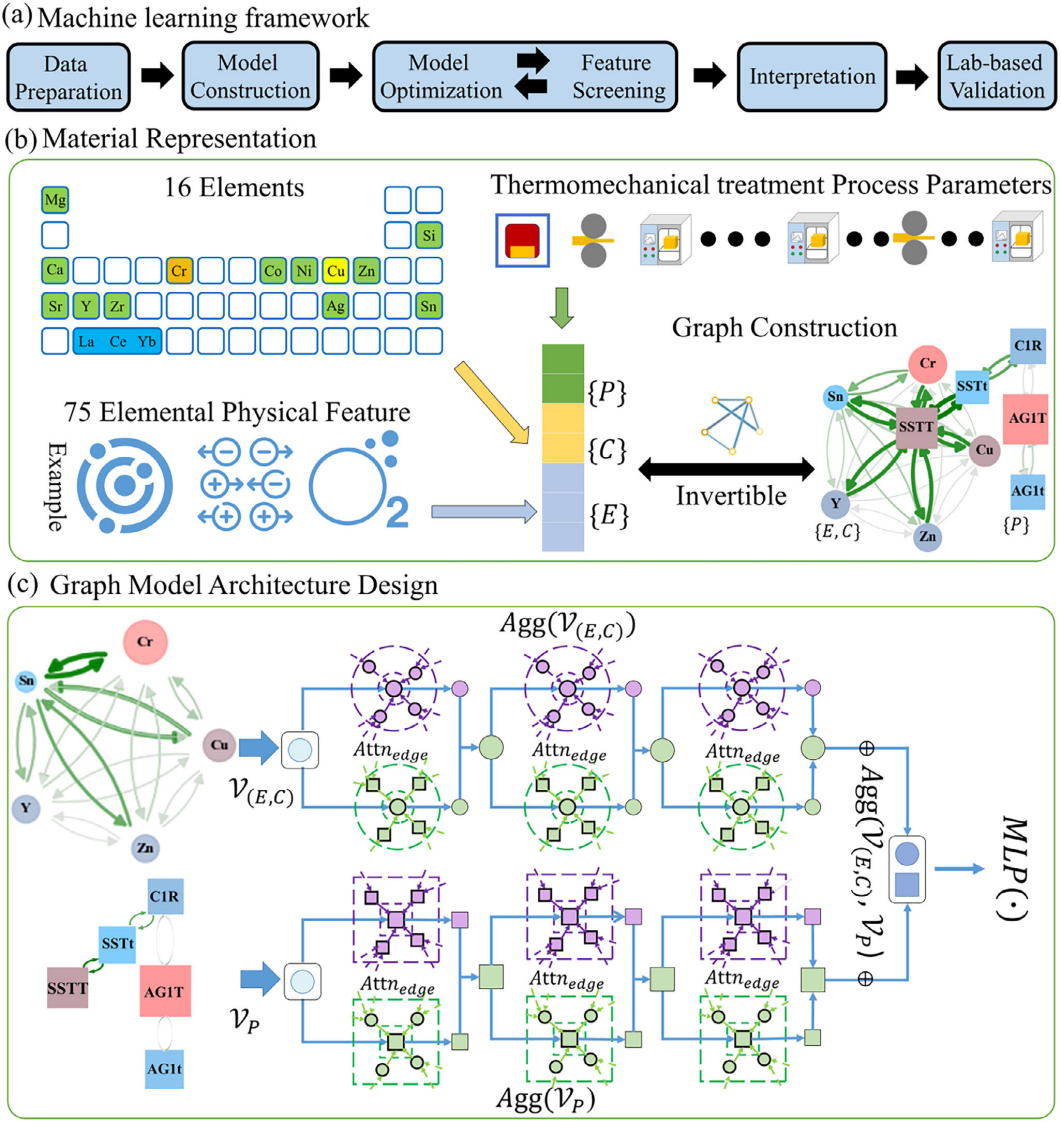
Workflow of the heterogeneous graph learning framework for alloy design. (a) The machine learning pipeline encompasses data preparation, model construction, feature screening through model optimization, interpretation of results, and subsequent experimental validation. (b) Material representation involves the integration of three data types: the elemental composition of the alloy (C), physical properties of the constituent elements (E), and process parameters (P). (c) The graph model architecture. The model includes two input vectors: V_(E,C)_, which denotes the vector containing the elemental composition and features, and V_P_, which denotes the vector containing process parameters. The TMT parameters consist of solid solution treatment temperature (SSTT), solid solution treatment time (SSTt), rolling deformation in the first pass (C1R), primary aging treatment temperature (AG1T), and primary aging treatment time (AG1t). These vectors are processed through attention (Attn) and aggregation (Agg) layers within a multilayer perceptron (MLP) to predict final material properties. The predicted outputs are the hardness and electrical conductivity of the alloy.

Herein, we describe a heterogeneous graph learning workflow illustrated in Figure 1 for overcoming these limitations. As shown in Figure 1a, the ML framework encompasses five parts: data preparation, model construction, model optimization and feature selection, model interpretation, and lab‐based validation. The initial procedure, data preparation, is detailed in Figure 1b. The collected data comprise three distinct components. The first is the chemical composition of the alloys, which includes 16 alloying elements. The second component consists of 75 fundamental physical properties for each element, such as Young's modulus, bulk modulus, and covalent radii. The third component is the process parameters for each alloy, including plastic deformation, and heat treatment processes. To ensure data quality, we implemented a dual‐cleaning strategy. First, we execute strict filtering to remove any samples with missing composition information or incomplete processing parameters. Sconed, statistical anomalies (identified via Interquartile Range and Z‐score) are subjected to secondary validation based on the physical metallurgy principles of each alloy system. Data points are only excluded if they are both statistically anomalous and violate the inherent, physically plausible property ranges of their respective alloy systems.

To create a unified and trainable data structure from the diverse alloy compositions and processing pathways, we transform the dataset into a heterogeneous graph. Within this graph, circular nodes represent the constituent elements of an alloy, and each node encapsulates 75 physical features in addition to the mass fraction of elements. Rectangular nodes denote the treatment processes and associated parameters. Bidirectional edges connect element nodes to represent potential interactions and also link element nodes to process nodes to model the influence of composition on processing. The graph structure is completed by directed edges that connect the process nodes in an ordered sequence to capture the procedural workflow and interdependent relationships. This study compiled over 1800 data points from 23 alloy systems, where alloys with identical elemental compositions were grouped as one system regardless of concentration variations. Since hardness data far exceeded yield strength records in our dataset, leveraging hardness data enhanced dataset utilization and model robustness. Crucially, for Cu alloys, hardness reliably correlates with yield strength [25], serving as an effective proxy. Therefore, hardness and electrical conductivity were selected as the target prediction outputs.

For model optimization and feature selection, we identify key features affecting alloy properties using correlation analysis, recursive elimination, recursive addition, and exhaustive screening. After an initial correlation screening, we apply the training results to further vet the features. Using prediction error as the criterion, recursive elimination progressively deletes unimportant features. To prevent the inadvertent removal of critical variables, recursive addition is used to build feature sets up from a minimal combination. Finally, exhaustive screening is employed on the resulting subset to select the feature combination that minimizes model prediction error.

In the model interpretation part, we analyze the key physical features retained after the selection process to understand their influence on alloy properties. This involves a comprehensive analysis of three distinct architectural components: the element nodes with features, the process nodes, and the connecting edges between them. The analysis of element and process node importance identifies the key elements and process that most strongly affect the properties. Concurrently, assessing the significance of the connecting edges reveals the critical interrelationships and interactions between these nodes.

For lab‐based validation, our model guided the design of both a specific alloy composition and its optimal processing pathway. The resulting purpose‐built alloy, Cu‐Cr‐Zr‐Y‐La‐Mg‐Zn, was synthesized according to the model‐prescribed process routes. The microstructures were characterized, and the properties were tested, validating our computational predictions.

### 2.2 Feature Selection and Interpretability

**FIGURE 2:**
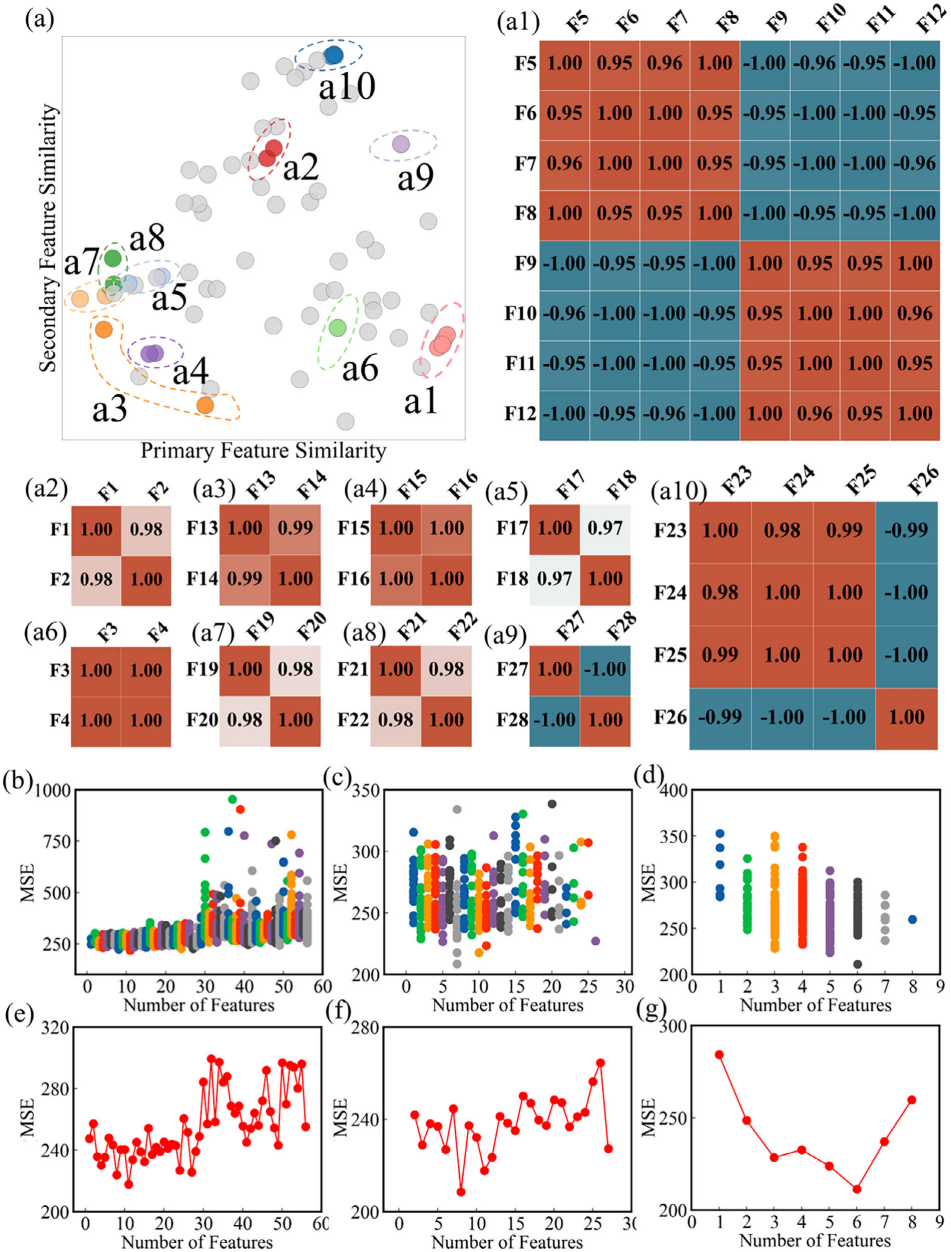
The feature selection process. (a) Correlation analysis of elemental physical features with a correlation coefficient greater than 0.95 are indicated by matching colors, while gray data points represent features without significant inter‐correlation, (a1–a10) correlation coefficient results corresponding to a1–a10. (b‐g) Variation of prediction error, measured as Mean Squared Error (MSE), during recursive feature elimination (b, c), recursive feature addition (d, e), and exhaustive screening (f, g).

To develop a robust predictive model, we initiated the feature selection process with a dataset comprising 75 distinct elemental physical features. An initial analysis revealed strong collinearity between descriptors, identifying ten subgroups with high correlation coefficients (>0.95), such as the compression modulus and bulk modulus (Figure 2a). Gray data points represent feature that are not significantly correlated with other features, whereas data points of the same color scheme indicate a high correlation between these features. Figure 2a1–a10 represents heat maps of correlation coefficients between highly correlated features in Figure 2a, respectively. To mitigate this multicollinearity, after correlation screening, the feature space was reduced to 57 unique descriptors (Tables S1 and S2). Subsequently, we employed a comprehensive strategy combining recursive feature elimination, addition, and exhaustive search methods to identify the most salient predictors (Figure 2b–g). The Mean Squared Error (MSE) was selected as the metric for evaluating model predictions. It quantifies the average squared deviation between the predicted values and the actual experimental results, and is calculated as follows:

$$MSE=\frac{1}{n}\sum_{i=1}^{n}{\left(y_{exp}-y_{pred}\right)}^{2}$$

where n is the number of samples, *y_exp_* represents the actual experimental results, and *y_pred_* represents the predicted results.

Figure 2b,e demonstrates the recursive feature elimination process, where a great reduction in model prediction error is observed as the number of features decreases. To prevent the accidental removal of critical features, we ultimately retained the 27 most representative features. Figure 2c,f displays the recursive feature addition results, with Figure 2f clearly indicating that the model achieves optimal predictive performance when the feature count is maintained at eight, reaching the global minimum prediction error at this point. We then systematically evaluated all possible feature combinations through exhaustive enumeration, ultimately identifying an optimal set of six features (Figure 2d,g). This multi‐stage process yielded a final parsimonious set of six features: atomic concentration, atomic environment number, electron affinity, mass attenuation coefficient, effective nuclear charge, and Young's modulus.

With this feature set established, we next sought to validate their importance and probe the evolution of their impact during training in Figure S1. Among the 23 alloy systems collected in this study, the Cu‐Cr‐Ag‐Zr alloy was selected for feature importance analysis at three distinct and representative stages: initial, middle, and terminal stages. Notably, in the terminal stage, the prediction error for electrical conductivity is approximately 15%. This is primarily because the electrical conductivity of copper alloys is extremely sensitive to lattice defects. Given that this study covers over 20 alloy systems and a variety of complex thermomechanical processing routes, the error actually falls within the reasonable range of measurement reproducibility across different laboratories. For hardness prediction, Young's modulus, mass attenuation coefficient, and electron affinity showed consistent positive effects, while Cu mass fraction remained detrimental. In contrast, feature impacts on conductivity evolved during training: initially, Cu mass fraction exhibited a counterintuitive negative correlation where higher copper content typically enhances conductivity [26, 27, 28], but the model later corrected this, ultimately showing its strong positive influence alongside negative correlations from Young's modulus and electron affinity. These results indicate that the model progressively learns meaningful feature‐property relationships beyond simple data fitting.

### 2.3 Model Interpretability Analysis and Rational Alloy Design Validation

**FIGURE 3:**
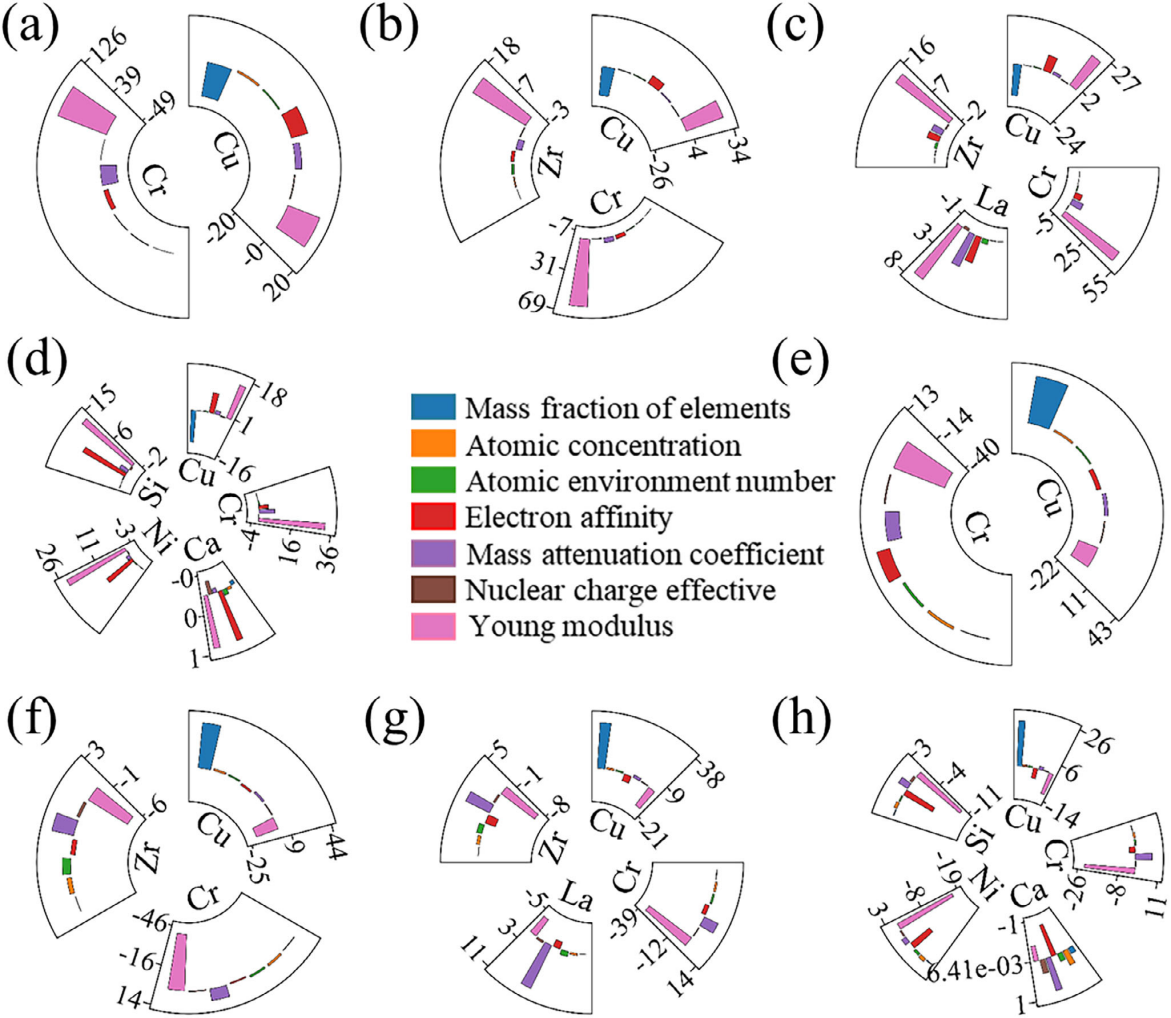
Feature importance analysis results in the different representative alloy systems. (a‐d) Analysis results of features influence on hardness, (a) Cu‐Cr (binary alloy), (b) Cu‐Cr‐Zr (ternary alloy), (c) Cu‐Cr‐La‐Zr (quaternary alloy), and (d) Cu‐Cr‐Ca‐Ni‐Si (quinary alloy). (e‐f) Analysis results of features influence on conductivity, (e) Cu‐Cr (binary alloy), (f) Cu‐Cr‐Zr (ternary alloy), (g) Cu‐Cr‐La‐Zr (quaternary alloy), and (h) Cu‐Cr‐Ca‐Ni‐Si (quinary alloy). A feature with positive importance has a beneficial effect on the alloy's properties, while one with negative importance has a detrimental effect. The extent of the effect increases with the absolute value of the importance.

Although the model has successfully identified the key features, it should be noted that the impact of different elemental features varies significantly in different alloy systems. To better understand the degree to which each feature affects performance, this study conducted a feature importance analysis for each of the 23 alloys systems in the collected dataset. For clarity of presentation, one representative system from each category (binary, ternary, quaternary, and quinary alloys) was selected for display in Figure 3. The complete set of results is provided in Figures S2 and S3.

Figure 3a–d presents the feature importance analysis regarding alloy hardness. The results indicate that the mass fraction of Cu, Young's modulus, Mass attenuation coefficient, and Electron affinity are the most critical factors affecting hardness. It is reasonably understood that in copper alloys, a higher mass fraction of Cu generally leads to lower hardness. The Young modulus reflects the strength of atomic bonds in material and the ability to resist elastic deformation. Higher Young modulus results in alloys with greater resistance to deformation and higher hardness. The mass attenuation coefficient can affect the length of metallic bonds in an alloy, which in turn affects the lattice distortion [29]. Since the mass attenuation coefficient of Cu element is low, the higher the mass attenuation coefficient of the added element, the more serious the lattice distortion in the alloy, and the higher the hardness of the alloy. The influence of electron affinity on hardness is contingent upon the formation of precipitates. When alloying elements, such as Ni and Si (Figure 3d), can form intermetallic precipitates (e.g., Ni_2_Si and Ni_3_Si) [3], a higher electron affinity promotes stronger, more directional bonding within these phases, resulting in harder precipitates that significantly enhance overall alloy hardness.

Figure 3e–h presents the feature importance analysis regarding the electrical conductivity of the alloys. All results consistently indicate that a higher mass fraction of Cu leads to greater electrical conductivity, which aligns well with theoretical expectations. Moreover, the Electron affinity and Young's modulus show a negative correlation with the electrical conductivity, and the Mass attenuation coefficient shows a positive correlation with the electrical conductivity. The Electron affinity represents the ability of the nucleus to attract electrons. An element with a higher electron affinity energy has a greater attraction to electrons, which is more likely to interrupt electron motion and reduce the concentration of free electrons in the alloy, thereby decreasing the electrical conductivity [29]. The alloying elements with higher Young's modulus result in greater lattice distortion in the matrix, greater ability to scatter electrons, and lower electrical conductivity of the alloy. A larger Mass attenuation coefficient of an alloying element means a larger number of electrons outside the nucleus of the atom, which contributes more to the free electron density of the alloy [2].

**FIGURE 4:**
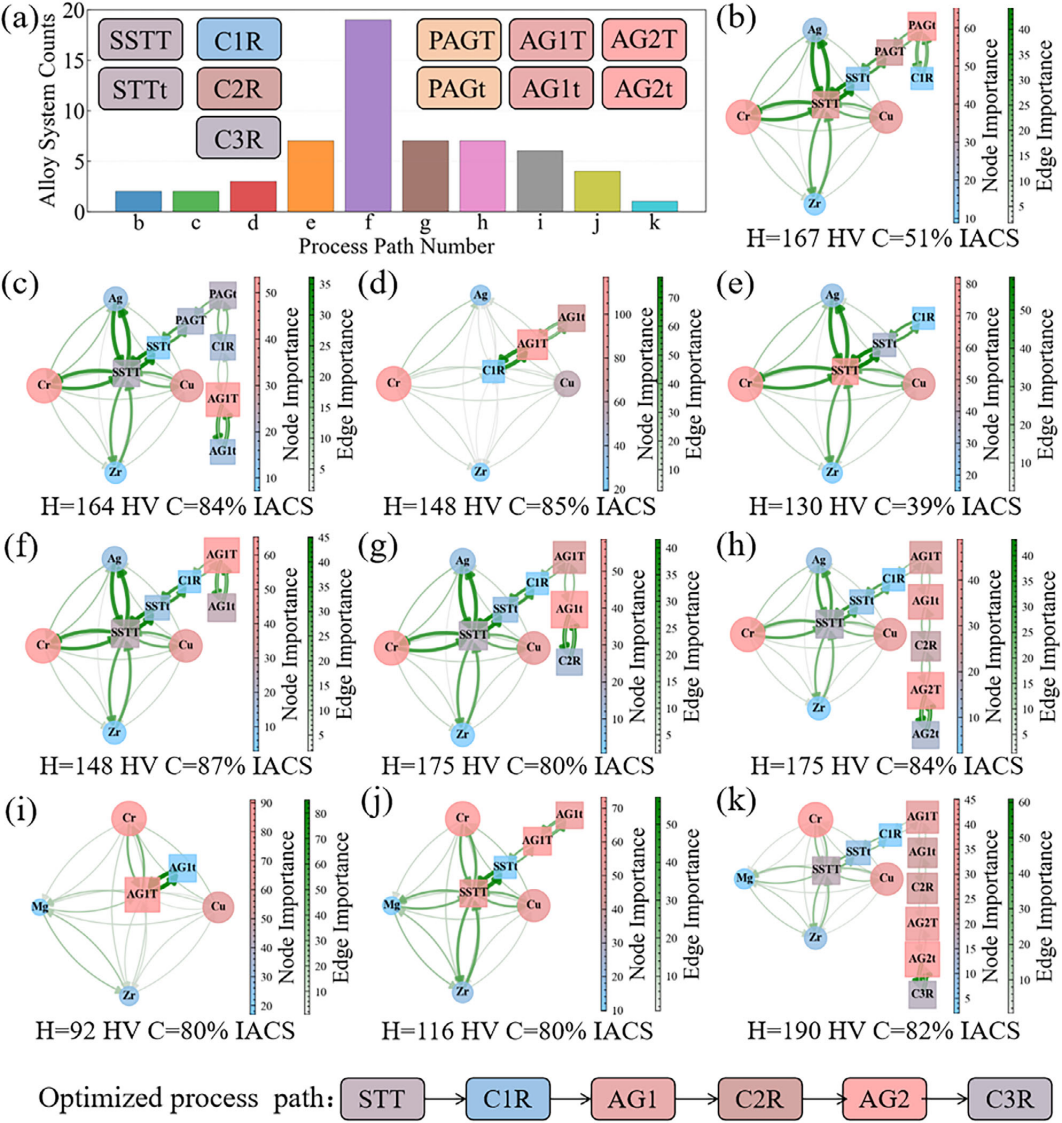
The effect of different processes on the alloy performance. (a) Process parameters and process routes are present in the alloy system of this study. SSTT (solid solution treatment temperature), SSTt (solid solution treatment time), C1R (first cold rolling), C2R (second cold rolling), C3R (third cold rolling), PAGT (Pre‐aging treatment temperature), PAGt (Pre‐aging treatment time), AG1T (first aging treatment temperature), AG1t (first aging treatment time), AG2T (second aging treatment temperature), AG2t (second aging treatment time). The English letters on the X‐axis represent the process paths in (b‐k). (b‐k) Importance analysis results under different process treatments: (b) SSTT‐ SSTt‐PAGT‐PAGt‐C1R, (c) SSTT‐ SSTt‐PAGT‐PAGt‐C1R‐AG1T, (d) C1R‐AG1T‐AG1t, (e) SSTT‐SSTt‐CR, (f) SSTT‐STTt‐C1R‐AG1T‐AG1t, (g) SSTT‐STTt‐C1R‐AG1T‐AG1t‐C2R, (h) SSTT‐STTt‐C1R‐AG1T‐AG1t‐C2R‐AG2T‐AG2t, (i) AG1T‐AG1t, (j) SSTT‐SSTt‐AG1T‐AG1t, (k) SSTT‐STTt‐C1R‐AG1T‐AG1t‐C2R‐ AG2T‐AG2t‐C3R. The abbreviations H, C, and IACS are defined as hardness, electrical conductivity, and the International Annealed Copper Standard, respectively.

After identifying the influence of features on alloy performance, this study further systematically investigates the effect of processes on performance regulation, with the results shown in Figure 4. Across all 23 alloy systems, there are 11 types of process parameters and 10 typical process paths (Figure 4a). Given the large total number of process combinations (63 in total) across different systems, two representative alloy systems were selected to clearly demonstrate the impact of the 10 process paths, with complete results provided in the Figures S4–S6.

This study systematically investigates the influence of TMT on alloy performance through two distinct yet complementary aspects: individual process parameters and integrated processing pathways. The results identify the following key governing parameters: first aging temperature and time, secondary cold rolling, secondary aging temperature and time, and third cold rolling. The optimal processing pathway is determined to be solution treatment followed by cold rolling and subsequent aging treatment. This route demonstrates consistent superiority over alternative sequences including direct aging treatment, cold rolling with aging treatment, solution treatment with aging treatment, and solution‐aging treatment ‐cold rolling. Furthermore, multi‐stage TMT yields enhanced properties relative to single‐stage TMT.

The results in Figure 4b–k indicates that aging temperature and time are the most critical process parameters affecting alloy properties. For example, comparing Figure 4b,c, after aging treatment, the hardness of the alloys is similar, but the electrical conductivity increases significantly by over 30% IACS (International Annealed Copper Standard). In contrast, Figure 4d–f indicates that the first cold rolling pass (C1R) is not the dominant factor in performance regulation, showing weak interactions with alloying elements. Particularly important is the comparison between Figure 4e,f, which shows that after C1R followed by aging treatment, both hardness and electrical conductivity improve significantly (from 130 HV, 39% IACS to 148 HV, 87% IACS), indicating that the aging treatment parameters have a far greater impact on properties than rolling.

Figure 4e–h shows that secondary rolling (C2R) and secondary aging treatment are also key process parameters. Comparing Figure 4f,g, after C2R, alloy hardness increases significantly while conductivity slightly decreases; after subsequent secondary aging treatment (Figure 4h), hardness remains stable and conductivity further increases. According to these results, secondary TMT is associated with improved performance. Figure 4k indicates that a third cold rolling (C3R) pass can also yield some performance benefits.

Furthermore, the results indicate that the processing sequence influences the final properties. Figure 4d–f,i,j shows a strong coupling effect between the solid solution treatment temperature (SSTT) and alloying elements. A comparison between Figure 4i,j shows that alloys subjected to solid solution treatment followed by aging treatment achieve a 24 HV higher hardness than those undergoing direct aging treatment. Consequently, solid solution treatment is an essential initial step in the alloy processing route.

Additionally, incorporating cold rolling between solution treatment and aging treatment leads to substantially improved comprehensive properties, elevating the performance from 116 HV, 80% IACS (Figure 4j) to 151 HV, 82% IACS (Figure S5k). Comparing Figure 4b,f, under the “aging before rolling” sequence, the alloy properties reach 167 HV, 51% IACS, while the “rolling before aging” sequence results in 148 HV, 87% IACS. The latter shows a hardness decrease of 19 HV but a substantial 35% IACS improvement in conductivity. Further comparing Figure 4c,g, alloys treated with secondary TMT (175 HV, 84% IACS) perform significantly better than those subjected to pre‐aging + TMT (164 HV, 84% IACS). In summary, adopting a “solution treatment + multi‐stage TMT” route (rolling before aging treatment) can effectively synergize and enhance the overall performance of the alloy.

**FIGURE 5:**
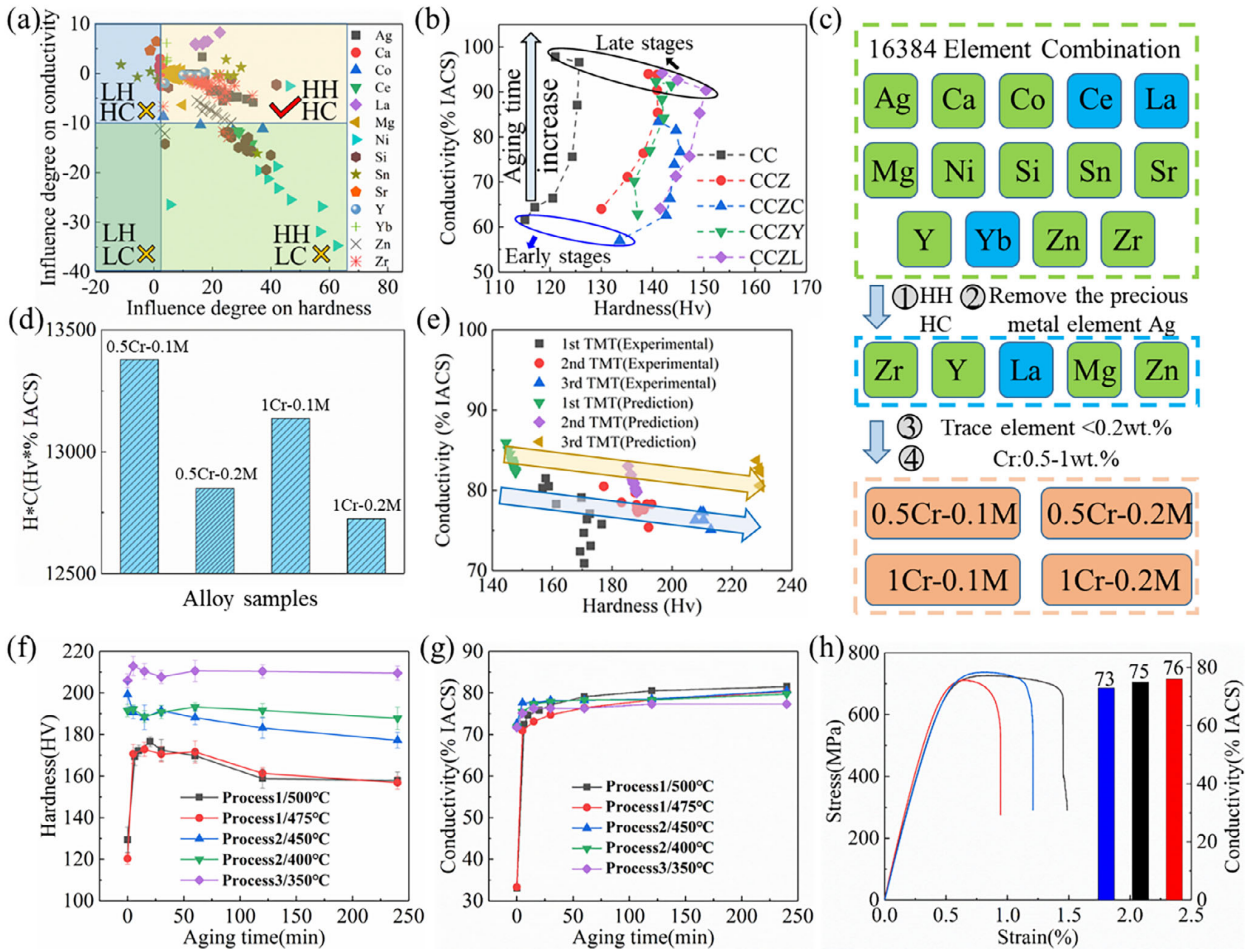
Interpretability analysis of the predictive model and experimental validation results. (a) The predicted influence of different alloying elements on achieving high hardness (HH), high conductivity (HC), low hardness (LH), or low conductivity (LC) in Cu‐Cr alloys. (b) Experimental performance data for various alloys subjected to different TMT routes. The alloys shown are Cu‐Cr (CC), Cu‐Cr‐Zr (CCZ), Cu‐Cr‐Zr‐Co (CCZC), Cu‐Cr‐Zr‐Y (CCZY), Cu‐Cr‐Zr‐La (CCZL)). (c) Alloy Design Strategy, M denotes combinations of Zr, Y, La, Mg, and Zn elements. For example, 0.1 M represents 0.1Zr‐0.1Y‐0.1La‐0.1Mg‐0.1 Zn (wt. %). (d) Comparison of H*C values for four different Alloys, where H means hardness, and C is conductivity. (e) Comparison of experimental results and model predictions for the complex Cu‐0.5Cr‐0.1 M alloy across various multi‐stage TMTs. (f–h) Performance change curves of alloy under multi‐stage heat treatment: (f) Aging curves showing the evolution of hardness as a function of time at various aging temperatures, (g) Corresponding aging curves showing the evolution of electrical conductivity under the same conditions. (h) Representative tensile stress‐strain curves for the alloy, with final electrical conductivity values indicated.

This study systematically summarizes the overall influence mechanisms of various elements on alloy properties, and through the preparation and testing of a series of novel alloys, validates the model's accuracy, as shown in Figure 5. The effects of various alloying additions on the hardness and electrical conductivity of Cu‐Cr alloys were mapped (Figure 5a). The related analysis results are shown in Figures S7 and S8 for details. This analysis identifies four distinct property regimes: high‐hardness, high‐conductivity (HH‐HC); high‐hardness, low‐conductivity (HH‐LC); low‐hardness, high‐conductivity (LH‐HC); and low‐hardness, low‐conductivity (LH‐LC). One group of elements (La, Y, Zr, Ag, Mg, and Zn) achieves the sought‐after HH‐HC state. In contrast, another group (Ni, Si, Co, and Ce) enhances hardness but severely compromises conductivity, placing these elements in the HH‐LC region. Elements such as Sr, Ca, and Yb fall into the LH‐HC category, improving conductivity while reducing hardness. Finally, Sn is unique in that it occupies multiple regimes, demonstrating that its role is highly context‐dependent and varies with the specific alloying system (see Figures S7 and S8).

Experimental validation of the model was performed by fabricating five alloys of distinct compositions (Cu‐Cr, Cu‐Cr‐Zr, Cu‐Cr‐Zr‐Co, Cu‐Cr‐Zr‐Y, and Cu‐Cr‐Zr‐La) and tracking their properties through an aging treatment (Figure 5b). Early‐stage results showed that adding Zr to the Cu‐Cr base improved both hardness and conductivity, while further additions of Y or La produced additional increases in hardness, likely by accelerating Cr precipitation. The addition of Co, in contrast, increased hardness but compromised conductivity. Through prolonged aging, the alloys containing Zr, Y, and La maintained high hardness with a minor drop in conductivity, whereas the Co‐containing alloy exhibited a persistent and substantial conductivity penalty. These experimental observations directly corroborate the property relationships identified by our model (Figure 5b), confirming its predictive power.

Leveraging the model's predictions, from the 16 384 possible combinations, we designed a new Cu‐Cr‐Zr‐Y‐La‐Mg‐Zn alloy engineered for cost‐effectiveness by omitting high‐cost Ag, as shown in Figure 5c. Then, we used the model to predict the properties of Cu‐0.5Cr‐xZr‐yY‐zLa‐wMg‐vZn alloys (with x, y, z, w, v taking values of 0.1, 0.2, 0.3, 0.4, and 0.5 wt. %, respectively), totaling 3125 compositional combinations. The model clearly revealed the trend that as copper content decreases, alloy hardness increases while conductivity declines (Figure S9a–d). Guided by these findings and the well‐recognized target in Cu‐Cr alloy of 700 MPa ultimate tensile strength and 70% IACS conductivity, we implemented two key compositional constraints to ensure performance [30]. First, we have strictly controlled the content of any single trace element below 0.2 wt. % in accordance with literature [3, 31], to guarantee the achievement of the 70% IACS conductivity benchmark. Second, given that the precipitation strengthening by nano‐sized Cr particles precipitated from the Cu matrix is the dominant strengthening mechanism, the Cr content was set in the range of 0.5–1 wt. %, guided by its maximum solid solubility (∼0.7 wt. %) in the Cu matrix [32, 33]. Therefore, we have fabricated four alloys Cu‐0.5Cr‐0.1 M, Cu‐1Cr‐0.1 M, Cu‐0.5Cr‐0.2 M, and Cu‐1Cr‐0.2 M), as shown in Figure 5c. Here, the collective symbol M denotes the elemental group comprising Zr, Y, La, Mg, and Zn. For instance, 0.1 M corresponds to a total addition of 0.1 wt. % for each element (i.e., 0.1Zr‐0.1Y‐0.1La‐0.1Mg‐0.1 Zn (wt. %)).

As shown in the Figure S9e–f, the experimental results for the four designed alloys are highly consistent with the trends predicted by the model. By comparing the hardness‐conductivity product (H × C value) of the four alloys, we found that the 0.1 M alloys outperformed the 0.2 M alloys, with Cu‐0.5Cr‐0.1 M achieving the highest H × C value (Figure 5d). Therefore, an optimized multi‐stage TMT process was then developed for this alloy, guided by the ML interpretability analysis. Both experimental results and model predictions indicate that the peak comprehensive performance is reached after the third TMT stage (Figure 5e). The alloy's performance evolution is shown in Figure 5f,g. These optimized processes (see Methods) yield significant gains in hardness with only a minimal conductivity penalty, resulting in a final material with a high yield strength of 700–725 MPa, an ultimate tensile strength of 710–740 MPa, and a remarkable electrical conductivity of 73%–76% IACS (Figure 5h).

### 2.4 Model Mechanistic Validation of Designed Alloys

**FIGURE 6:**
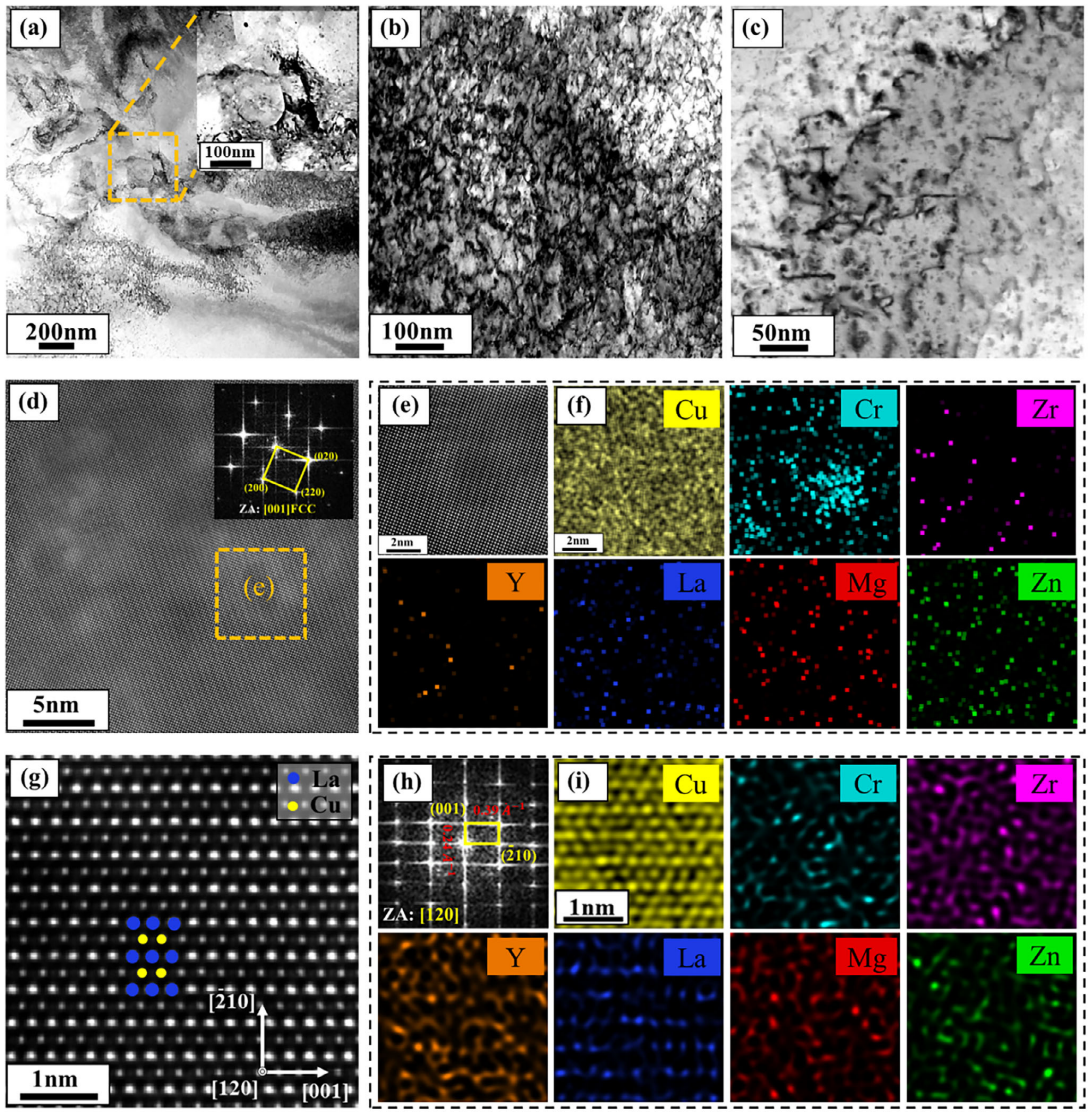
Microstructural characterization of the Cu‐Cr‐Zr‐Y‐La‐Mg‐Zn alloy. (a) Bright‐field transmission electron microscopy (TEM) image showing a partially recrystallized structure containing fine grains (inset). (b) Bright‐field TEM image revealing a high density of dislocations within the matrix. (c) Magnified bright‐field TEM image showing a high number density of nanoprecipitates. (d) High‐angle annular dark‐field (HAADF) scanning transmission electron microscopy (STEM) image with its corresponding fast Fourier transform (FFT) pattern (inset). The pattern indicates that the nanoprecipitates possess a face‐centered cubic (FCC) structure, are coherent with the matrix, and have a diameter of 2–3 nm. (e) High‐resolution HAADF‐STEM image of the region marked in (d) with (f) corresponding energy‐dispersive X‐ray spectroscopy (EDS) elemental maps. The maps show that the nanoprecipitates are rich in Cr, identifying as a typical type of Cr‐rich precipitate in Cu alloys. (g) High‐resolution HAADF‐STEM image with its corresponding FFT pattern (h). (i) EDS elemental maps corresponding to (g). Based on the elemental maps showing Cu, Y, and La enrichment within the nanoprecipitates and the supporting FFT results, the precipitated phase is determined to be Cu_5_(Y, La).

To elucidate the origin of the superior performance in the model‐optimized Cu‐Cr‐Zr‐Y‐La‐Mg‐Zn alloy, we characterized the microstructure using TEM. The analysis reveals a hierarchical microstructure (Figure 6a–c) comprising fine localized recrystallized grains (Figure 6a) and unrecrystallized zones with high‐density dislocation networks (Figure 6b). Throughout the matrix, we observe homogeneously dispersed nanoscale precipitates (Figure 6c). Figure 6d–i shows the presence of two different precipitation phases in the matrix. HAADF‐STEM and the corresponding FFT pattern (Figure 6d) indicate that the first phase consists of ultra‐fine precipitates, approximately 2–3 nm in size, which maintain full coherency with the face‐centered cubic (FCC) matrix. Energy‐dispersive X‐ray spectroscopy (EDS) analysis (Figure 6e,f) confirms these nanoprecipitates are Cr‐rich, a finding consistent with the typical Cr precipitates widely reported in conventional Cu‐Cr‐Zr based alloys [34, 35]. The second precipitated phase, shown in the high‐resolution image (Figure 6g), exhibits a distinct atomic arrangement. The corresponding FFT pattern (Figure 6h) reveals a hexagonal crystal structure. EDS analysis (Figure 6i) confirms that these second nanoprecipitates contain Cu, La, and Y elements. Collectively, these crystallographic and compositional results indicate the presence of the phase Cu_5_(Y, La) [36]. Notably, the identification of Cu_5_(Y, La) constitutes a new discovery within the processing space of Cu‐Cr based alloys.

**FIGURE 7:**
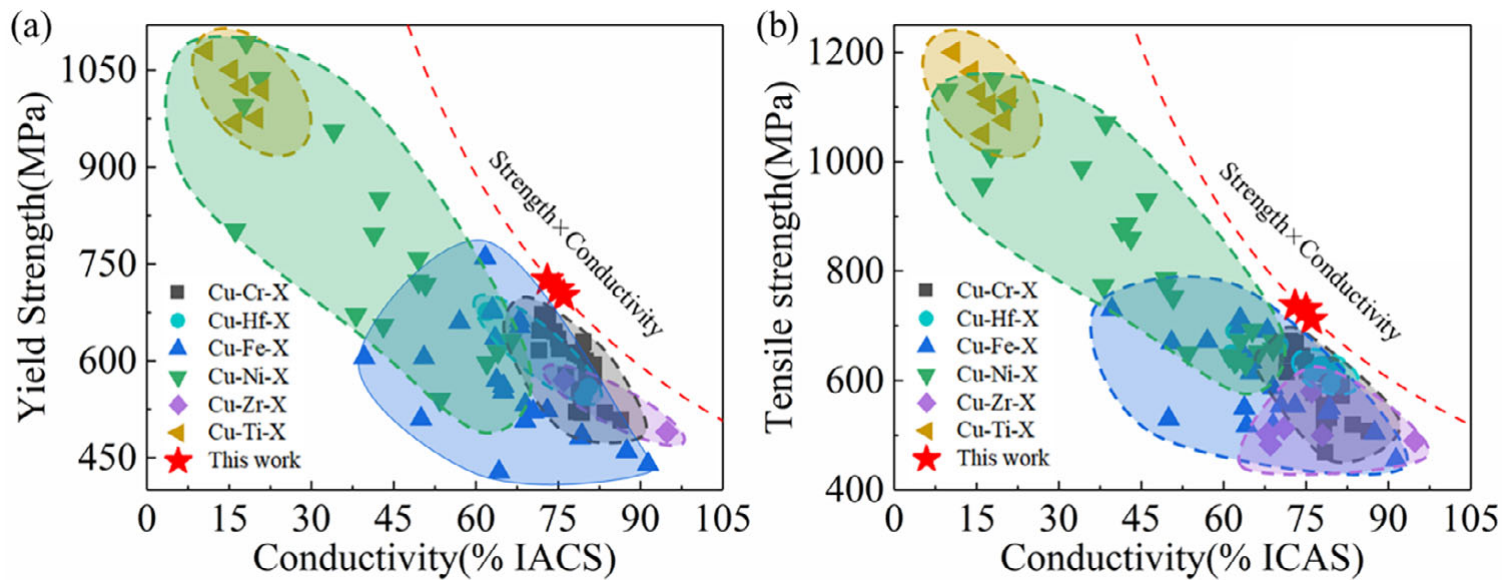
Strength‐conductivity properties of the Cu‐Cr‐Zr‐Y‐La‐Mg‐Zn alloy. A comparison of the strength and electrical conductivity of the alloy developed in this study against a broad range of Cu alloys prepared via conventional TMT routes. (a) Yield strength and conductivity, (b) Tensile strength and conductivity. The comparison data include Cu‐Cr‐X alloy [34, 35, 38, 39, 42, 43, 44, 45, 46, 47, 48, 49, 50, 51, 52, 53, 54, 55, 56, 57, 58, 59, 60, 61, 62, 63, 64, 65, 66, 67], Cu‐Hf‐X alloy [13, 68, 69], Cu‐Fe‐X alloy [70, 71, 72, 73, 74, 75, 76, 77], Cu‐Ni‐X alloy [78, 79, 80, 81, 82, 83, 84, 85], Cu‐Zr‐X alloy [40, 86, 87], Cu‐Ti‐X [88, 89, 90, 91, 92], where X denotes other trace elements contained in the alloy. The performance data for commercial Cu alloys is sourced from Wieland Company [93].

Figure 6d demonstrates that this approach successfully produced ultrafine Cr precipitates (2–3 nm), which are approximately 50% smaller than previously reported values (>5 nm) [37, 38]. More notably, Y, La, and Cu co‐formed a novel nanoscale strengthening phase, Cu_5_(Y, La), with a size distribution in the tens of nanometers range (Figure S10). The controlled nanoscale precipitates act synergistically with refined recrystallized grains (Figure 6a) and high‐density dislocation networks (Figure 6b), collectively establishing exceptional mechanical properties through the combined strengthening effects of precipitation, grain boundary, and dislocation mechanisms [3, 39, 40, 41]. The highly refined precipitate structure significantly enhances conductivity through the solute depletion zone mechanism [3, 28]. Ultimately, this developed Cu‐Cr‐Zr‐Y‐La‐Mg‐Zn alloy achieved an outstanding combination of yield strength (710 MPa), tensile strength (726 MPa), and electrical conductivity (75% IACS) (Figure 7), with its comprehensive performance significantly surpassing existing Cu‐based alloy systems via conventional TMT routes.

While existing ML methods exhibit strong data modeling capabilities, they face significant bottlenecks when handling the inherent heterogeneous variability of alloy compositions and processing paths. Constrained by the requirement for fixed‐structure data inputs, traditional models primarily rely on two strategies—data deletion or zero‐padding—to cope with variable pathways. Both approaches have notable drawbacks. Data deletion retains only samples with identical processing paths, discarding a substantial amount of valuable data and undermining statistical power. Conversely, zero‐padding attempts to align variable‐length sequences into fixed‐length vectors, constructing a sparse, high‐dimensional input space that exacerbates the curse of dimensionality. More critically, this mathematical convenience often obscures the underlying physical reality, making it difficult for traditional methods to capture high‐order dynamic interactions (element‐element, element‐process, and process‐process).

This study introduces an HGNN that integrates alloy compositions and processing paths into a unified graph structure, encoding processing sequences as directed graphs (e.g., solution treatment → cold rolling → aging). Compared to conventional approaches that rely on fixed‐length embeddings to force alignment, our model preserves fundamental distinctions in node types (alloy element nodes vs. process step nodes) and variable connection structures (e.g., the dynamic addition, deletion, or reordering of heat treatment sequences). By explicitly modeling topological order and capturing physically meaningful differences in processing histories, our approach expands the limits previously set by fixed‐path models, retaining all 1800+ heterogeneous samples for training while preserving the physical authenticity of the data. Building on this foundation, the present work incorporates interpretability analysis to extract actionable composition‐processing relationships, culminating in an end‐to‐end experimental validation that directly links model predictions to successfully fabricated alloys.

To rigorously evaluate our model's ability to capture physical evolution laws, we benchmarked our framework against eight common ML baselines (BRR, ElasticNet, SVR, GBR, RF, DTR, KNN, and ANN) as well as an advanced sequence‐learning model (LSTM), all trained on the identical dataset. The results demonstrate that while the eight standard tabular models generally suffer from low predictive accuracy, the advanced LSTM sequence model also exhibits critical limitations. Although the LSTM performs reasonably well in hardness prediction, its error margin for electrical conductivity remains substantial, indicating a struggle to capture the inherent physical trade‐offs in copper alloys. More importantly, the predicted property trajectories of both the standard baselines and the LSTM deviate markedly from experimental measurements. In several instances, they exhibit trends that completely violate fundamental physical laws—such as erroneously predicting an increase in electrical conductivity with progressive thermomechanical processing. In stark contrast, our HGNN accurately reproduces the physically consistent evolution of both properties across sequential processing steps. This comparative analysis demonstrates that our model systematically learns and respects the fundamental physical laws of TMT—a capability absent in traditional baseline approaches.

Guided by this HGNN, we successfully identified the governing physical features that dictate the performance of Cu alloys and quantitatively decoded the individual and synergistic effects of elemental composition and process parameters. The model revealed that the critical trade‐off between hardness and electrical conductivity is controlled by the complex interplay of three fundamental material descriptors: Young's modulus, mass attenuation coefficient, and electron affinity. Specifically, high Young's modulus enhances hardness by promoting stronger atomic bonding and suppressing precipitate coarsening, but concurrently degrades conductivity through increased lattice distortion. The mass attenuation coefficient exhibits a dual role: it intensifies lattice distortion to improve hardness while also contributing to conductivity by supplying additional free electrons. In contrast, electron affinity consistently degrades electrical conductivity by reducing the concentration of free carriers.

Critically, the model demonstrates that these effects are not merely additive or linear but interact through a complex, process‐dependent synergy. For example, we demonstrate that in binary Cu‐Cr alloys, Cr exhibits a significantly greater strengthening effect than Cu, despite having a moderately higher Young's modulus (Figure 3a). Furthermore, this influence is not constant across systems: the strengthening contribution of Cr's Young's modulus is notably weakened in Cu‐Cr‐Zr alloys (Figure 3b), revealing a clear element‐element interaction. To comprehensively decode these intricate property‐governing relationships, our model explicitly integrated element‐element, element‐process, and process‐process interactions. Finally, a comprehensive mapping of elemental effects (Figure 5a) identifies six alloy elements within the high‐hardness‐high‐conductivity (HHHC) region. After excluding the precious metal Ag, our analysis pinpointed five key alloying elements for this system: Zr, Y, La, Mg, and Zn. To ensure the robustness of these predictive capabilities, the model was systematically validated across nine distinct copper alloy compositional systems (Figure 5b–d), spanning ternary, quaternary, and trace‐element‐doped spaces. The performance evolution trends of all nine alloys are in excellent agreement with the model's predictions. This comprehensive validation fully demonstrates the framework's predictive accuracy and broad generalization capability across diverse compositional spaces, extending well beyond the single optimized application target. Finally, this novel combination was strategically selected to leverage a simultaneous combination of high Young's modulus (Zr, Zn), high mass attenuation coefficients (La, Y, Zr), and consistently low electron affinity across all five elements, ultimately forming the high‐performance Cu‐Cr‐Zr‐Y‐La‐Mg‐Zn alloy.

Beyond compositional design, the model quantifies the influence of processing parameters and deciphers complex element‐process interactions (Figure 4). It identified solution temperature, aging temperature, aging time, and rolling deformation as the most critical control factors. The model also revealed specific interactions: for instance, significant coupling exists between the SSTT and alloying elements (Figure 4b), whereas C1R shows no such coupling (Figure 4d). Furthermore, the model showed that processing pathways are critical. A pre‐aging treatment had a limited impact on properties (Figure 4c), while a multi‐stage TMT significantly influenced overall performance (Figure 4h). This comprehensive analysis allowed us to optimize the final processing route, selecting a high‐temperature solid solution treatment followed by a multi‐stage TMT. Based on these insights, we implemented an optimized protocol for the Cu‐Cr‐Zr‐Y‐La‐Mg‐Zn alloy.

In high‐performance Cu alloy systems, nanoscale precipitates constitute the fundamental structural units that govern the complex trade‐off between strength and electrical conductivity [9, 10, 29]. To maximize the number of strengthening phases, this model employed an integrated process combining high‐temperature solid solution treatment with multi‐stage TMT. The initial high‐temperature solid solution treatment ensured complete dissolution and atomic‐scale homogeneous distribution of Cr atoms within the copper matrix. Subsequently, a multi‐stage TMT was applied, which not only provided the necessary nucleation driving force to significantly increase the number density of Cr precipitates but also simultaneously promoted the formation of refined recrystallized grains and high‐density dislocation networks. Crucially, the key alloying elements (Zr, Y, La, Mg, Zn), leveraging their high Young's modulus and mass attenuation coefficients, introduced substantial lattice distortion into the Cu matrix. This distortion effectively reduced the atomic diffusion rates of Cr, which is the primary mechanism responsible for inhibiting precipitate coarsening and thus preserving the ultra‐fine microstructure necessary for high strength. Additionally, the co‐formation of the Cu_5_(Y, La) phase further contributed to strengthening. These multi‐scale structural features collectively enable exceptional mechanical properties.

Regarding conductivity, the multi‐stage TMT facilitates the full precipitation of solute atoms. This process is key, as it minimizes electron scattering by solutes and increases the mean free path of free electrons [9, 28]. The resulting highly refined precipitate structure and solute‐depleted matrix are thus directly responsible for the significant improvement in electrical conductivity. Simultaneously, the low electron affinity of the added elements maintains free electron concentration, while La, Y, and Zr further contribute additional free electrons via their high mass attenuation coefficients, collectively ensuring high electrical conductivity.

Collectively, these results demonstrate that multi‐scale microstructural coordination, guided by a unified heterogeneous graph network approach, can effectively overcome typical performance trade‐offs in alloy design. Despite these promising capabilities, several important directions remain to be explored. In future work, to further validate the practical value and generalization capability of our HGNN model, we will systematically evaluate its cross‐domain predictive accuracy across different processing sequences, novel processing steps, and diverse alloy composition systems. In parallel, we will integrate this framework into an iterative closed‐loop optimization system using active learning or Bayesian optimization, aiming to establish an autonomous “prediction–validation–feedback–optimization” cycle. This will facilitate a shift in materials discovery from passive analysis toward active design. Ultimately, these efforts will highlight the practical value of our HGNN model in accelerating next‐generation high‐performance materials development, offering a viable pathway from composition discovery and process design to performance optimization.

## 3 Conclusion

In summary, we have developed an HGNN that addresses the dimensional complexity and data sparsity inherent in modeling dynamic TMTs. Leveraging the HGNN, we identified Young's modulus, mass attenuation coefficient, and electron affinity as the three key physical descriptors governing the strength‐conductivity trade‐off in Cu alloys. This approach decoded critical composition‐processing relationships, enabling the strategic co‐design of a novel Cu‐Cr‐Zr‐Y‐La‐Mg‐Zn alloy and its optimized multi‐stage TMT route. Experimental validation confirmed the formation of highly desirable multi‐scale microstructural features, including ultra‐fine Cr nanoprecipitates (2–3 nm) and a novel strengthening Cu_5_(Y, La) phase, complemented by refined grains. Through the synergistic effects of precipitation, grain boundary, and dislocation strengthening, this alloy achieves an exceptional combination of yield strength (710 MPa), tensile strength (726 MPa), and conductivity (75% IACS). This study demonstrates that applying HGNN to the combined representation of alloy composition and processing pathways provides an effective strategy for accelerating the development of next‐generation high‐performance materials.

## 4 Methods

### 4.1 Data Structure

For model training, we constructed a heterogeneous graph for each alloy, comprising two distinct node types and three edge types. The “composition” node encapsulates the mass fractions of the constituent elements alongside 75 elemental features. The “process” node represents the complete process history of the alloy, including all manufacturing and treatment steps. To connect these nodes, we defined three types of edges: “element‐element” edges linking the elemental components, “element‐process” edges connecting compositional elements to processing steps, and “process‐process” edges linking sequential processing steps. The resulting metadata structure for our model is defined as: Metadata = ([“elements,” “process”], [(“elements,” “e,” “elements”), (“elements,” “e_p,” “process”), (“process,” “p,” “process”), (“process,” “rev_e_p,” “elements”)]). To facilitate understanding, Figure S11 presents an illustrative example of the conversion from raw data to heterogeneous graph data, including the corresponding computational data format.

### 4.2 Model Architecture

Our heterogeneous GNN model is composed of three interconnected modules. The initial module is a feature extractor based on a GNN comprising three Graph Attention Convolution (GATConv) layers with 128, 128, and 32 neurons in the hidden layers, respectively. This architecture transforms the varied compositions and processing histories of the alloys into fixed‐dimension node features. The second module, the aggregation module (G_aggr), pools the features from all nodes within the heterogeneous graph to generate a comprehensive global feature vector. This global vector is subsequently mapped to the target output dimension through a linear layer. The final module, the connection module (G_process), integrates the feature extraction and aggregation modules, enabling an end‐to‐end workflow from heterogeneous graph data input to feature extraction, aggregation, and ultimately, prediction output.

The model was trained using the Adam optimizer with an initial learning rate of 1 × 10^−4^. We employed a ReduceLROnPlateau learning rate scheduler, which dynamically adjusts the learning rate by a factor of 0.5 if the validation loss (min mode) did not improve for two consecutive epochs. The MSE (MSELoss, with reduction = “mean”) was used as the loss function. During the feature screening, which involved correlation analysis, recursive elimination, recursive addition, and exhaustive screening, each model was initially pre‐trained until a significant reduction in training error was observed. This was followed by a final training phase of 50 epochs, with the test set error serving as the criterion for feature selection. Our feature selection process, schematized in Figure S12, began with 57 features (after correlation analysis). The pre‐training and final training results are detailed in Figures S13–S16.

### 4.3 Comprehensive Evaluation of Model Performance: Baseline Comparison, Feature Selection, and Robustness Analysis

To comprehensively evaluate the performance of the proposed model, we conducted systematic analyses in the Supporting Information, with a focus on three aspects: (i) comparison with baseline models, (ii) effectiveness of the feature selection strategy, and (iii) robustness of the selected features under different random seeds. Detailed discussions are provided in Pages 22–29 of the Supplementary Information, with corresponding results illustrated in Figures S17–S20.

Specifically, we first trained ten commonly used models on the same dataset as baselines‐including Bayesian Ridge Regression (BRR), Linear Regression (LR), ElasticNet, Support Vector Regression (SVR), Gradient Boosting Regression (GBR), Random Forest (RF), Decision Tree Regression (DTR), k‐Nearest Neighbors (KNN), and Artificial Neural Networks (ANN)‐to validate the superiority of our proposed model. Next, we compared the effectiveness of three feature selection strategies: the method adopted in this study, the approach based on SHAP analysis, and the method based on the genetic algorithm. Finally, we systematically assessed the robustness of the selected features by training the model multiple times with different random seeds.

### 4.4 Alloy Fabrication

The alloy was synthesized from raw materials, including pure Cu, Cu‐10Cr (wt.%), Cu‐40Zr (wt.%), Cu‐15Y (wt.%), pure Mg, pure Zn, and pure La, using a vacuum induction melting furnace. To mitigate oxidation, all melting and casting were conducted in a strictly isolated environment. Before melting commenced, the furnace chamber underwent a rigorous “evacuation‐backfill” gas purging cycle: the chamber was evacuated to a high vacuum and then backfilled with high‐purity argon (Ar). This cycle was repeated three times to maximally expel ambient oxygen and establish a stable, protective atmosphere. The chemical composition of the fabricated alloy was determined by inductively coupled plasma‐atomic emission spectrometry (ICP‐AES) to be Cu‐0.48Cr‐0.04Zr‐0.07Y‐0.11La‐0.11Mg‐0.10 Zn (wt.%). The resulting as‐cast ingots were homogenized at 1000°C for 4 h. A 15 mm thick rectangular sample was sectioned from the ingot by the wire cutting. The samples then underwent a multi‐step TMT processing route consisting of: cold rolling by 50%, aging at 500°C for 20 min, a second cold rolling by 50%, aging at 400°C for 1 h, a cold rolling by 80%, aging at 350°C for 2 h. Subsequently, these samples were subjected to different final cold rolling deformations of 50%, 60%, and 70%, respectively.

### 4.5 Performance Testing

Electrical conductivity was measured using a D60K eddy current conductivity meter on samples with dimensions of 16 mm × 16 mm. The standard unit for measuring electrical conductivity is the International Annealed Copper Standard (IACS). Electrical resistance was measured with a QJ36C DC resistance bridge on 150 mm × 3 mm × 0.2 mm samples. For mechanical characterization, tensile specimens were machined into a dog‐bone shape with a gauge length of 26 mm, a width of 3 mm, and a thickness of 0.2 mm. Uniaxial tensile tests were performed at room temperature using an electronic universal testing machine. For each condition, three specimens were tested to ensure reproducibility.

### 4.6 Microstructure Characterization

Transmission electron microscopy (TEM) foils were prepared by mechanically grinding samples to a thickness of approximately 50 µm, followed by final thinning with an ion beam. Bright‐field TEM imaging was conducted using a Talos F200X microscope operating at 200 kV. High‐angle annular dark‐field (HAADF) scanning transmission electron microscopy (STEM) image, High‐resolution TEM (HRTEM) image, fast Fourier transform (FFT) analysis, and energy‐dispersive X‐ray spectroscopy (EDS) mapping were performed on a Thermo Fisher Spectra 300 microscope operated at 300 kV.

## Author Contributions

Jie Yin, Qian Lei, and Zhangwei Wang conceived the project. Jie Yin prepared the materials and conducted the TEM characterization. Jie Yin, Shuang Zhou, and Chong Wang performed the performance tests. Jie Yin, Yue Li, Wei Yan, Tong Xie, Bram Hoex, Qiang Long, Min Song, and Zhou Li contributed to the data analysis. Jie Yin, Qian Lei, Tong Xie, and Zhangwei Wang wrote the manuscript. Caoyang Jiang participated in data analysis. All authors contributed to the discussion of the results and commented on the manuscript.

## Conflicts of Interest

The authors declare no conflicts of interest.

## Supporting information




**Supporting File**: advs76392‐sup‐0001‐SuppMat.pdf.

## Data Availability

The data that support the findings of this study are available from the corresponding author upon reasonable request. Data and the source code can be accessed through: https://github.com/freshman‐124816/heterogeneous‐graph‐neural‐network.
