# Supplementary material for: Unifying Composition and Process Design: A Heterogeneous Graph Neural Network for Discovering High‐Performance Cu Alloys
Source: Adv Sci (Weinh). 2026 Jun 26:e24364. Online ahead of print. doi: 10.1002/advs.202524364 (PMC13336456; doi:10.1002/advs.202524364)
Supplement: Supplementary file 1 — Supporting File: advs76392‐sup‐0001‐SuppMat.pdf. [file ADVS-9999-e24364-s001.pdf]

Unifying Composition and Process Design: A Heterogeneous Graph Neural Network for Discovering High-Performance Cu Alloys

Jie Yin[1], Qian Lei[1,*], Yue Li[2], Tong Xie[3, 4,*], Shuang Zhou[1], Chong Wang[1], Bram Hoex[3, 4], Qiang Long[1], Caoyang Jiang[5], Min Song[1], Zhou Li[6], Zhangwei Wang[1,*]

1. State Key Laboratory of Powder Metallurgy, Central South University, Changsha 410083, China
2. Max Planck Institute for Sustainable Materials, Düsseldorf, 40237, Germany
3. University of New South Wales, School of Photovoltaics and Renewable Energy Engineering, UNSW Sydney, NSW 2052, Australia
4. Green Dynamics, Sydney, NSW 2052, Australia
5. The School of Automation, Central South University, Changsha 410083, China
6. School of Materials Science and Engineering, Central South University, Changsha 410083, China

* Corresponding author. Email: leiqian@csu.edu.cn; tong.xie@unsw.edu.au; z.wang@csu.edu.cn.

This PDF file includes:

Supplementary Fig. 1 to 16

Supplementary Table 1 to 2

Supplementary Table 1 Correspondence between feature numbers and names

Feature Number	Feature name
1	Atomic electron scattering factor at 0.5
2	Atomic concentration
3	Atomic environment number
4	chemical potential Miedema
5	Charge nuclear effective
6	Compression modulus
7	Conductivity electrical
8	Conductivity thermal
9	Debye temperature(K)
10	Density
11	Distance from core electron (Schubert)(A)
12	Distance from valence electron (Schubert)(A)
13	E1 electronegativity (Martynov&Batsanov)
14	Electronegativity (Pauling)
15	Electronegativity (Alfred-Rochow)
16	Electronegativity absolute
17	Energy of ionization first
18	Energy of ionization second
19	energy ionization third
20	Electrochemical weight equivalent
21	Electron affinity(kJ*mol-1)
22	Energy cohesive (Brewer)
23	Enthalpy of melting
24	Enthalpy of vacancies (Miedema)
25	Enthalpy of vaporization
26	Entropy of solid
27	Linear thermal expansion coefficient
28	Mass attenuation coefficient for MoKα
29	mass attenuation coefficient CrKalpha
30	Mass attenuation coefficient for CuKα
31	Magnetic resonance
32	Mass attenuation coefficient FeKa
33	Melting temperature
34	Mendeleev chemists sequence
35	Mendeleev d-t start left
36	Mendeleev Pettifor
37	Mendeleev Pettifor regular
38	Molar heat capacity
39	Moment nuclear magnetic
40	nW1/3 (Miedema)
41	nuclear charge effective Slater
42	Oxidation state first

43	Periodic number start counting top right. right-left sequence
44	Poisson ratio
45	Quantum number
46	Radii covalent
47	Radii metal (Waber)
48	Radii pseudo-potential (Zunger)
49	Resistivity electrical
50	Spectral lines no
51	Spin nuclei
52	Surface tension at Tm
53	Thermal neutron capture cross section
54	Valence electron number
55	work function
56	Volume of atom (Villars, Daams)
57	Young modulus

Supplementary Table 2 Elemental physical properties

Feature	Cu	Ag	Cr	Ca	Co	Ce	La	Mg	Ni	Si	Sn	Sr	Y	Yb	Zn	Zr
1	1.45	2.13	1.25	1.53	1.37	1.57	2.48	0.288	1.41	0.316	2.24	1.8	1.84	2.91	1.49	1.88
2	8.45	5.85	8.33	5.1	8.97	4.42	2.7	4.3	9.14	5	3.62	1.7	3.02	3.02	6.55	4.29
3	12	12	14	4	12	4	12	12	12	4	3	12	12	12	12	12
4	4.45	4.35	4.65	4.1	5.1	4.55	3.17	3.45	5.2	4.7	4.15	2.4	3.2	3.22	4.1	3.45
5	5.84	8.03	5.13	6.22	5.58	6.78	9.31	3.31	5.71	4.29	9.1	6.07	6.26	8.59	5.97	6.45
6	1.37	1.007	1.901	0.569	1.914	0.772	0.243	0.354	1.86	0.988	1.11	0.116	0.366	0.133	0.598	0.833
7	58.8	62	7.7	7.35	15.4	1.54	1.77	25	13.3	2.52E-10	8.85	5	1.54	3.7	16.4	2
8	401	429	93.7	40.6	100	59.9	13.5	156	90.7	148	66.6	49	17.2	34.9	116	22.7
9	343	225	630	320	445	374	142	400	450	645	200	147	280	120	327	291
10	8960	10500	7190	5907	8900	5323	6145	1738	8902	2329	7310	2540	4469	6965	7133	6506
11	1.12	1.3	0.84	1.38	0.71	1.22	1.34	1.09	1.13	1.18	1.41	1.39	1.22	1	1.25	0.98
12	2.55	2.9	1.33	2	1.14	1.99	2.42	2.3	2.9	1.91	2.3	2.92	2.42	1.99	2.21	1.96
13	1.08	1.07	2	1.7	1.72	1.99	1.35	1.31	1.76	1.98	1.88	1.13	1.41	1.1	1.44	1.7
14	1.9	1.9	1.6	1.6	1.9	1.8	1	1.2	1.9	1.8	1.8	1	1.2	1.05	1.6	1.4
15	1.75	1.42	1.56	1.82	1.7	2.02	1.08	1.23	1.75	1.74	1.72	0.99	1.11	1.06	1.66	1.22
16	4.48	4.44	3.72	3.2	4.3	4.6	3.1	8.3	4.4	4.77	4.3	2	3.19	3.5	4.45	3.64
17	745.4	731	652.8	578.8	758.4	762.1	538.1	737.7	736.7	786.5	708.6	549.5	615.6	603.4	906.4	660
18	1958	2074	1592	1979	1646	1537	1067	1451	1753	1577	1412	1064	1181	1174	1733	1267
19	3554	3361	2987	2963	3232	3302	1851	7733	3393	3232	2943	4207	1980	2408	3833	2218
20	0.32938	0.11793	0.08983	0.24083	0.30539	0.18808	0.47986	0.12601	0.30409	0.07269	0.30751	0.45404	0.30715	0.59772	0.33876	0.23632
21	118.5	125.7	64.3	30	63.8	116	50	-21	156	133.6	116	-146	29.6	50	9	41.1
22	3.49	2.95	4.1	2.81	4.39	3.85	4.47	1.51	4.44	4.63	3.14	1.72	4.37	1.6	1.35	6.25
23	13	11.1	21	5.59	16.3	36.8	10	8.9	17.2	50.6	7.07	8.2	11.5	9.2	7.28	19.2
24	100	90	120	48	135	55	120	50	130	60	50	63	125	65	50	170
25	306.7	257.7	341.8	256	382.4	330	402.1	127.6	374.8	383.3	296.2	154.4	367.4	159	114.2	566.7
26	33.3688	42.70536	23.86476	41.11438	30.06122	31.10792	56.94048	32.69891	29.89375	18.9662	51.45577	52.335	46.0548	62.802	41.65866	38.89537
27	16.6	19	4.5	18.3	13.1	5.8	12.1	25	13.5	2.6	22.2	22.5	11.3	25.2	30.3	5.7
28	50.9	25.8	31.1	60.1	42.5	64.8	45.8	4.11	46.6	6.44	31.1	95	100	84.5	55.4	13.9
29	166	586	79.8	212	125	235	202	121	144	189	662	373	396	401	189	419
30	53	210	260	55.9	313	75.6	341	38.6	45.7	60.6	256	125	134	146	60.3	145
31	11.285	1.9808	2.4063	10.2188	10.072	1.485	6.014	2.606	3.8048	8.458	15.868	1.8451	2.086	7.456	2.663	3.9578
32	103	391	463	131	76.8	146	557	74.8	88.6	117	451	236	252	265	117	265
33	1356	1234	2130	303	1768	1211	1193	922	1726	1683	505	1043	1796	1097	693	2125
34	16	15	60	76	69	80	23	13	72	90	79	11	24	51	18	55
35	69	68	54	75	63	80	15	12	66	81	79	10	16	43	72	47
36	72	71	57	81	64	84	33	73	67	85	83	15	25	17	76	49
37	72	71	57	78	66	83	34	18	69	84	82	16	48	21	75	50
38	24.435	25.351	23.35	25.86	24.81	23.347	27.11	24.89	26.07	20	26.99	26.4	26.53	26.74	25.4	25.36
39	2.221	-0.113	0.474	2.011	4.639	0.877	2.761	0.855	0.746	0.555	-1.041	1.089	0.137	-0.677	0.874	1.298
40	1.47	1.36	1.73	1.31	1.75	1.37	1.18	1.17	1.75	1.5	1.24	0.84	1.21	1.23	1.32	1.41
41	4.2	4.2	3.35	5	3.9	5.65	2.85	2.85	4.05	4.15	5.65	2.85	3	2.85	4.35	3
42	1	1	2	3	2	2	3	2	2	4	2	2	3	2	2	4
43	26	44	31	24	28	23	84	17	27	15	41	53	52	71	25	51
44	0.343	0.367	0.21	0.47	0.32	0.32	0.28	0.291	0.312	0.42	0.357	0.28	0.265	0.207	0.249	0.38
45	4	5	4	4	4	4	6	3	4	3	5	5	5	6	4	5
46	117	134	118	125	116	122	169	136	115	117	140	192	162	170	125	145
47	1.278	1.445	1.36	1.411	1.252	1.369	1.877	1.602	1.246	1.319	1.623	2.151	1.801	1.74	1.394	1.602
48	2.04	2.375	2.44	1.695	2.02	1.56	3.08	2.03	2.18	1.42	1.88	3.21	2.94	3.59	1.88	2.825
49	1.673	1.59	12.7	27	6.24	460000	57	4.45	6.84	100000	11.1	23	57	29	5.916	40
50	353	145	1133	55	920	62	293	98	505	157	131	92	98	159	115	134
51	1.5	0.5	1.5	1.5	3.5	4.5	3.5	2.5	1.5	0.5	0.5	4.5	0.5	2.5	2.5	2.5
52	1825	1250	2300	1100	2550	1000	1020	760	2450	1250	675	410	1125	500	990	2000
53	3.78	63.6	3.1	2.9	37.2	2.2	8.98	0.063	37.2	171	0.63	1.2	1.28	35	1.1	0.184
54	11	11	6	3	9	4	3	2	10	4	4	2	3	3	12	4
55	4.6	4	4.5	4	5	4.8	3.5	3.7	5.1	4.8	4.4	2.4	3.1	2.6	4.2	4
56	1.181	1.706	1.2	1.947	1.108	2.264	3.741	2.324	1.093	2.002	2.705	5.632	3.318	4.128	1.521	2.328
57	129.8	82.7	279	9.8	211	79.9	37.9	44.7	199.5	113	49.9	15.7	66.3	23.9	104.5	98

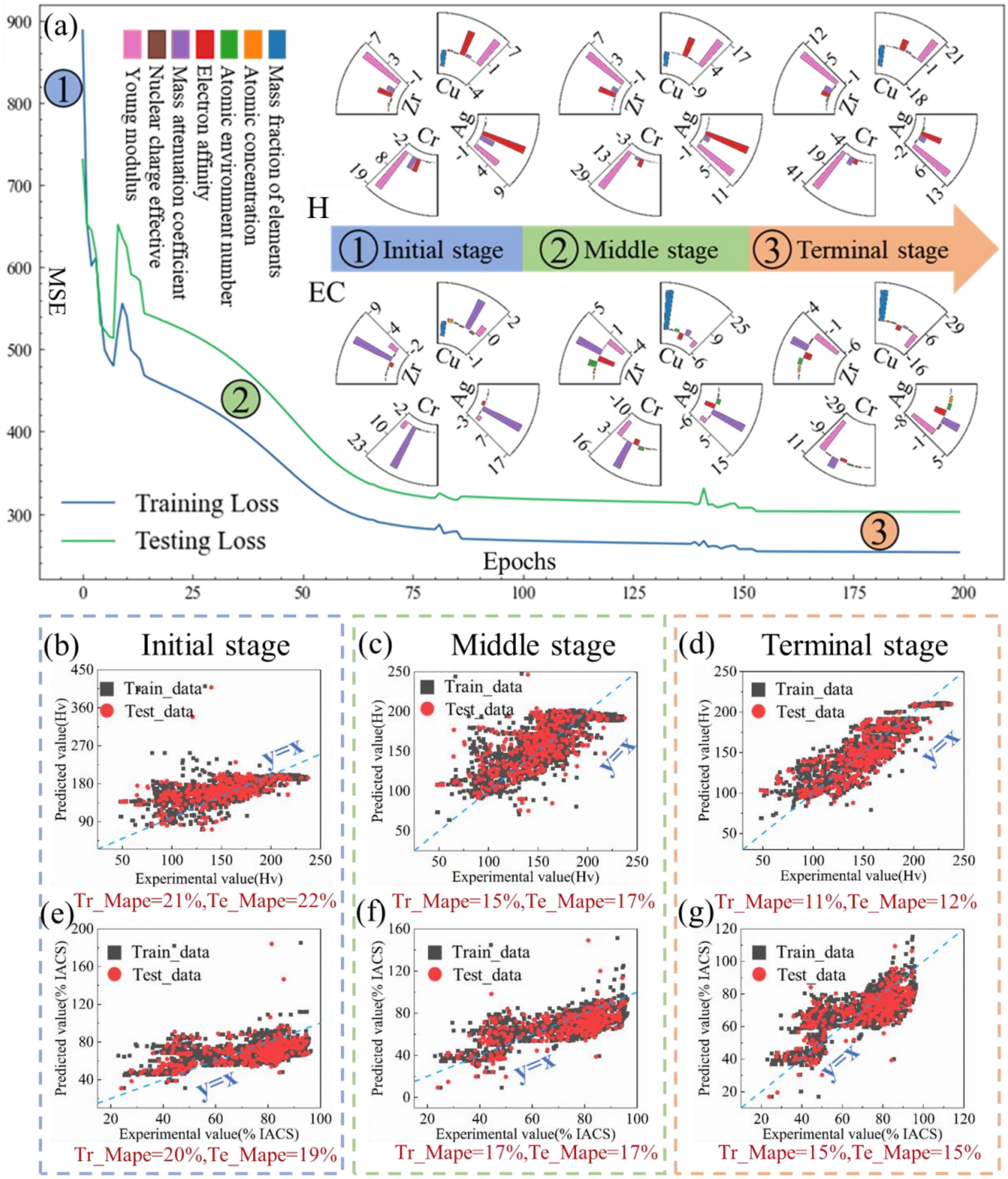

Supplementary Fig. 1 The influence variation of the selected features on performance prediction evaluated at different stages of model training. (a) Feature importance analysis at three distinct stages:① initial stage (high prediction error), ② middle stage (rapidly decreasing error) and ③ terminal satge (low, stable error). H means hardness, EC is electrical conductivity. MSE represents mean square error. **(b-g)** Comparison of model prediction results and experimental results of different stages: **(b, e)** Initial stage, **(c, f)** Middle stage, **(d, g)** Terminal stage. Tr_Mape means the mean absolute percentage error of train data. Te_Mape is the mean absolute percentage error of test data.

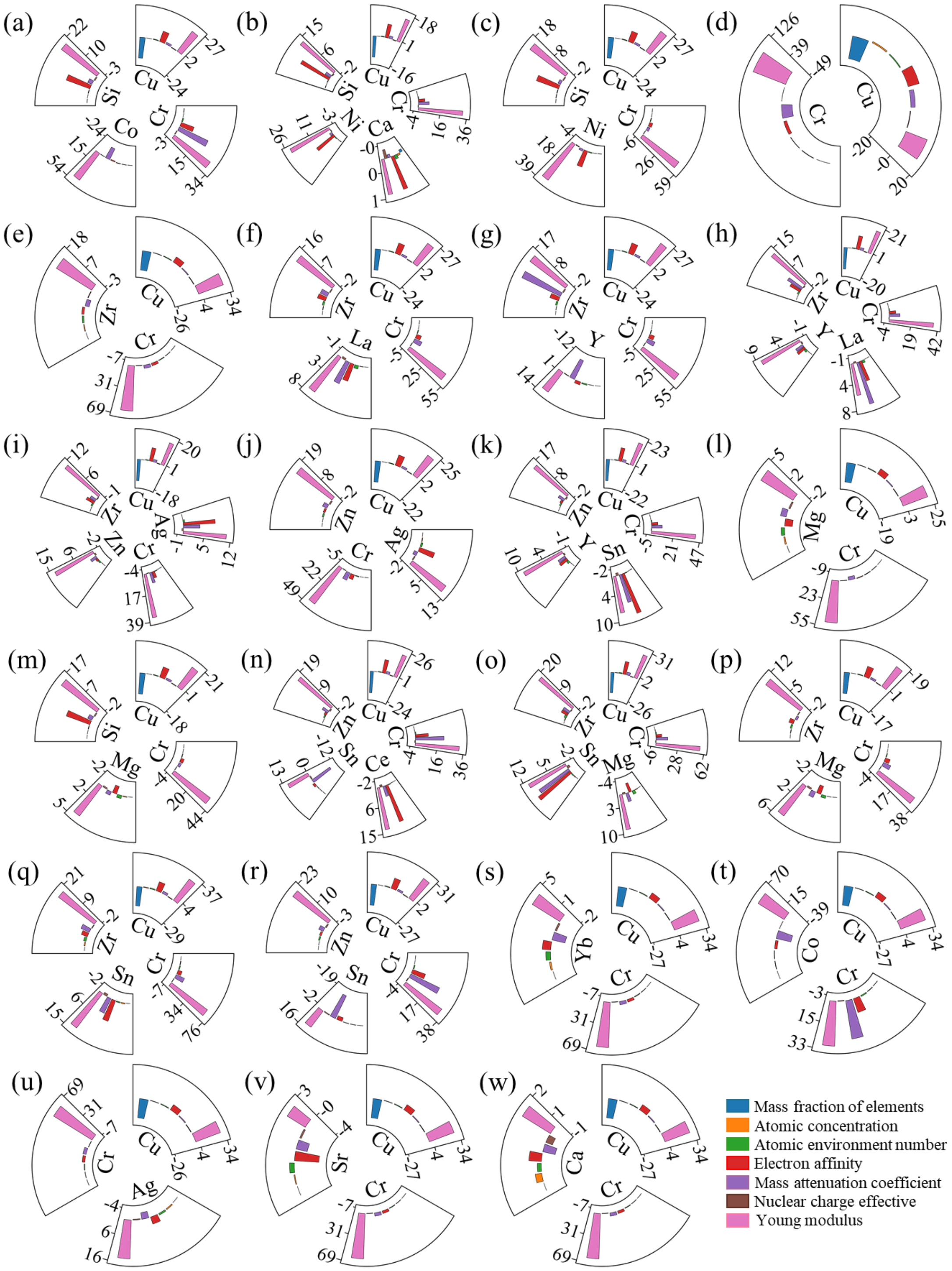

Supplementary Fig. 2 Feature importance analysis for hardness prediction in various Cu-based alloys. Each panel displays the analysis for a specific alloy composition as follows: (a) Cu-Cr-Co-Si, (b) Cu-Cr-Ca-Ni-Si, (c) Cu-Cr-Ni-Si, (d) Cu-Cr, (e) Cu-Cr-Zr, (f) Cu-Cr-La-Zr, (g) Cu-Cr-Y-Zr, (h) Cu-Cr-La-Y-Zr, (i) Cu-Ag-Cr-Zn-Zr, (j) Cr-Ag-Cr-Zn, (k) Cu-Cr-Sn-Y-Zn, (l) Cu-Cr-Mg, (m) Cu-Cr-Mg-Si, (n) Cu-Cr-Ce-Sn-Zn, (o) Cu-Cr-Mg-Sn-Zr, (p) Cu-Cr-Mg-Zr, (q) Cu-Cr-Sn-Zr, (r) Cu-Cr-Sn-Zn, (s) Cu-Cr-Yb, (t) Cu-Cr-Co, (u) Cu-Cr-Ag, (v) Cu-Cr-Sr, (w) Cu-Cr-Ca.

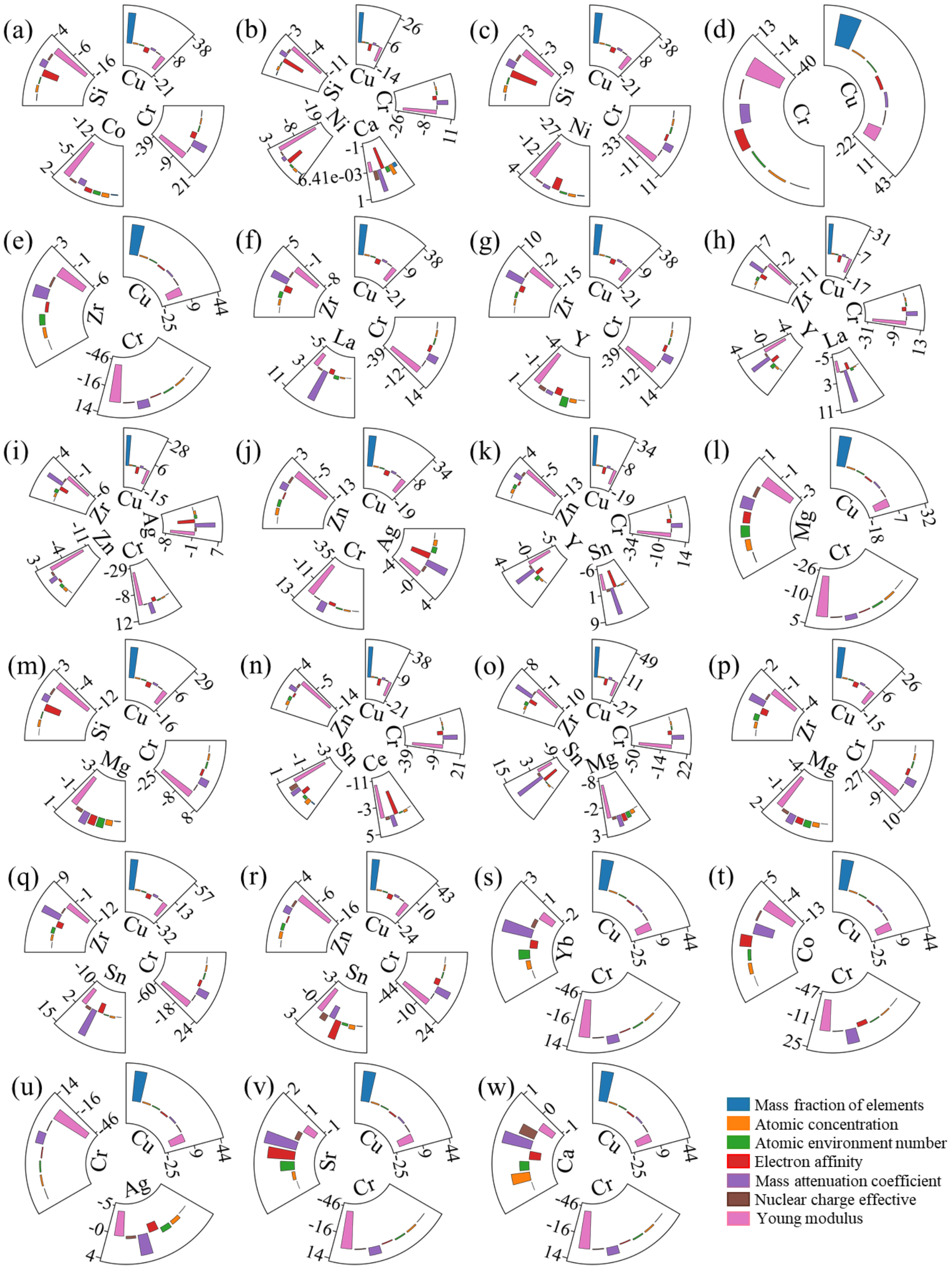

Supplementary Fig. 3 Feature importance analysis for conductivity prediction in various Cu-based alloys. Each panel displays the analysis for a specific alloy composition as follows: (a) Cu-Cr-Co-Si, (b) Cu-Cr-Ca-Ni-Si, (c) Cu-Cr-Ni-Si, (d) Cu-Cr, (e) Cu-Cr-Zr, (f) Cu-Cr-La-Zr, (g) Cu-Cr-Y-Zr, (h) Cu-Cr-La-Y-Zr, (i) Cu-Ag-Cr-Zn-Zr, (j) Cr-Ag-Cr-Zn, (k) Cu-Cr-Sn-Y-Zn, (l) Cu-Cr-Mg, (m) Cu-Cr-Mg-Si, (n) Cu-Cr-Ce-Sn-Zn, (o) Cu-Cr-Mg-Sn-Zr, (p) Cu-Cr-Mg-Zr, (q) Cu-Cr-Sn-Zr, (r) Cu-Cr-Sn-Zn, (s) Cu-Cr-Yb, (t) Cu-Cr-Co, (u) Cu-Cr-Ag, (v) Cu-Cr-Sr, (w) Cu-Cr-Ca.

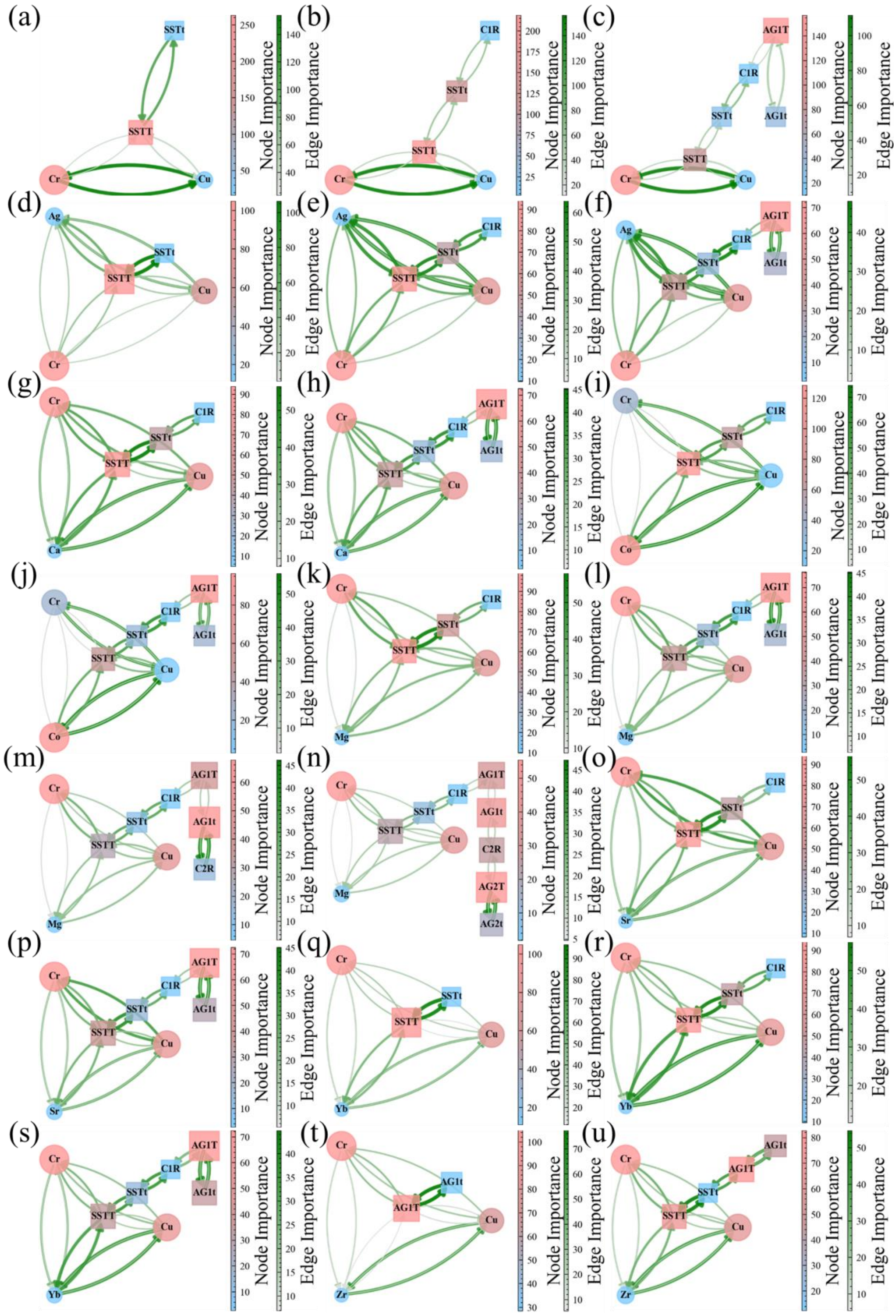

Supplementary Fig. 4 Importance Analysis of Composition and Process for Binary and Ternary Copper Alloys. (a-c) Cu-Cr, (d-f) Cu-Cr-Ag, (g-h) Cu-Cr-Ca, (f-j) Cu-Cr-Co, (k-n) Cu-Cr-Mg, (o-p) Cu-Cr-Sr, (q-s) Cu-Cr-Yb, (t-u) Cu-Cr-Zr

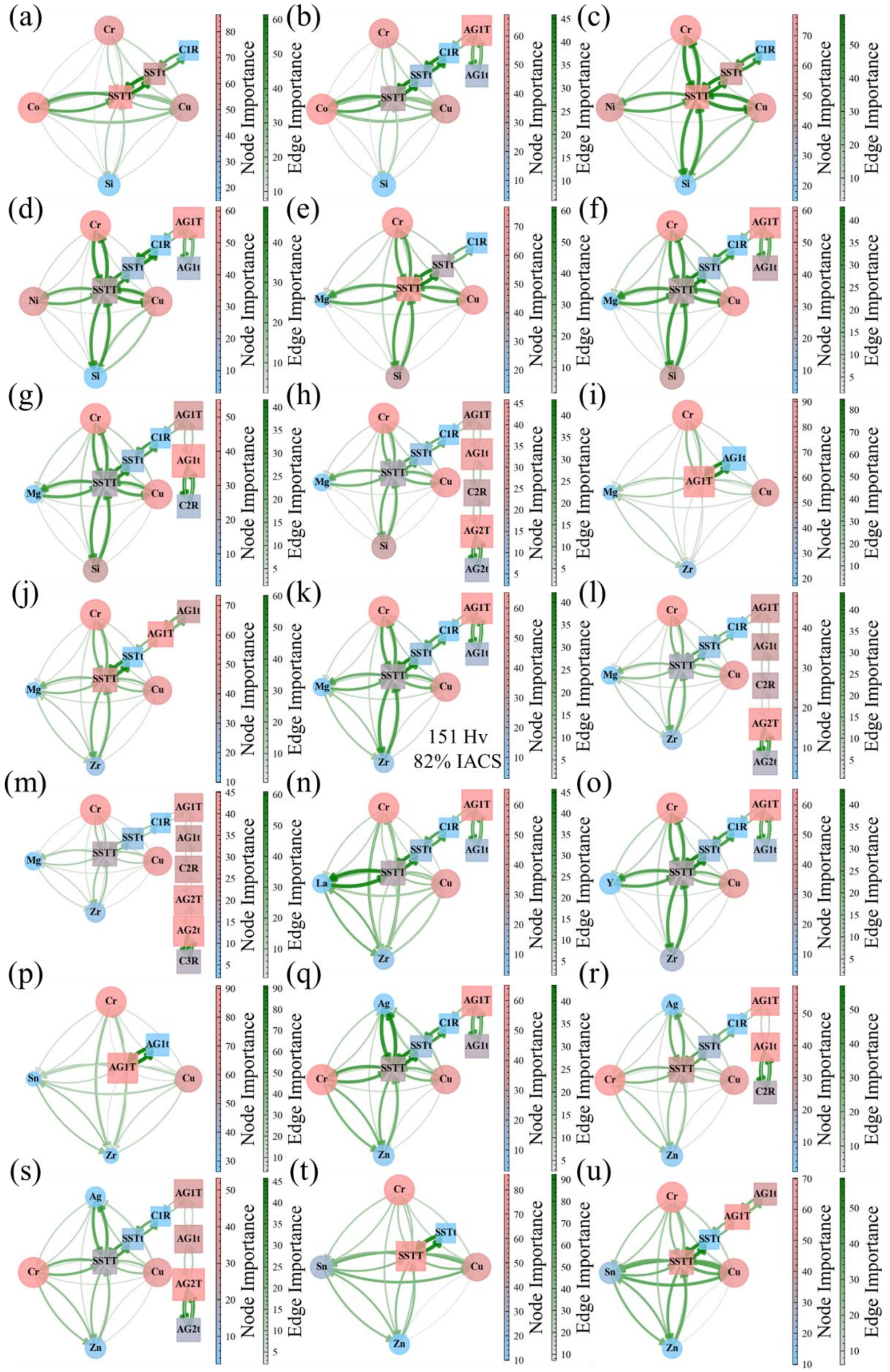

Supplementary Fig. 5 Importance Analysis of Composition and Process in Quaternary Copper Alloys. (a-b) Cu-Cr-Co-Si, (c-d) Cu-Cr-Ni-Si, (e-h) Cu-Cr-Mg-Si, (i-m) Cu-Cr-Mg-Zr, (n) Cu-Cr-La-Zr, (o) Cu-Cr-Y-Zr, (p) Cu-Cr-Sn-Zr, (q-s) Cu-Ag-Cr-Zn, (t) Cu-Cr-Sn-Zn.

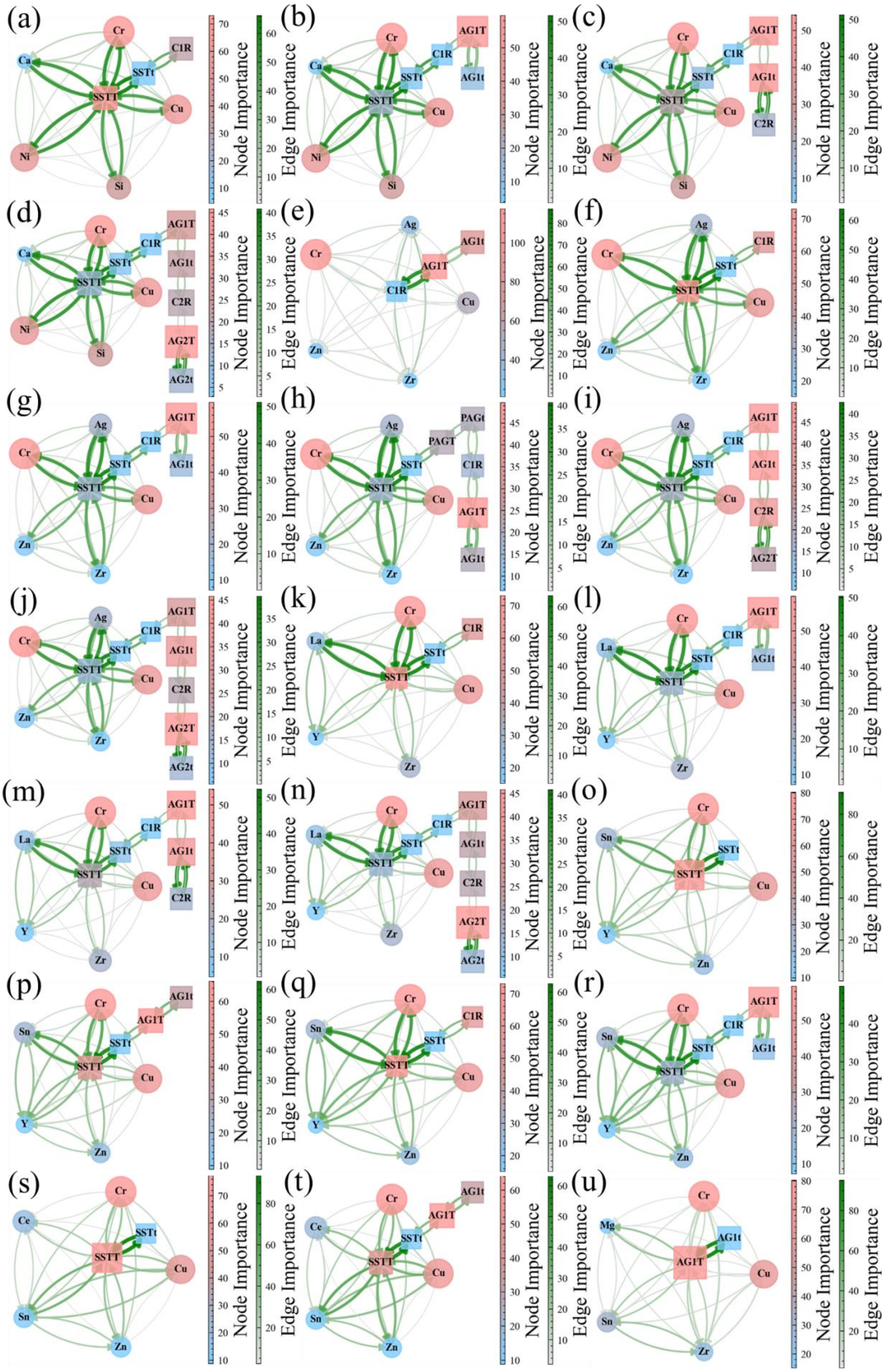

Supplementary Fig. 6 Importance Analysis of Composition and Process for Quintuple Copper Alloy. (a-d) Cu-Cr-Ca-Ni-Si, (e-j) Cu-Ag-Cr-Zn-Zr, (k-n) Cu-Cr-La-Y-Zr, (o-r) Cu-Cr-Sn-Y-Zn, (s-t) Cu-Cr-Ce-Sn-Zn, (u) Cu-Cr-Mg-Sn-Zr.

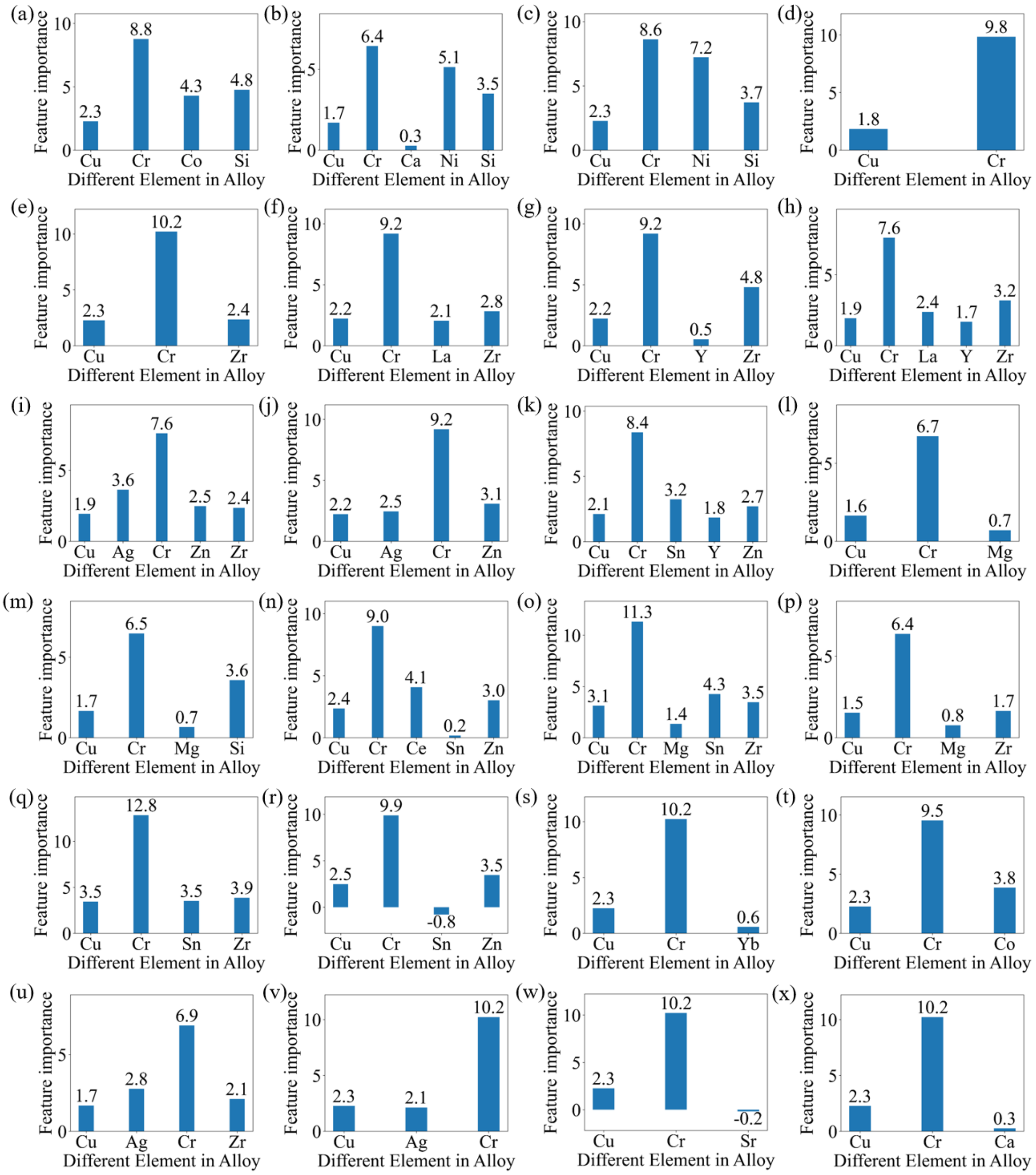

Supplementary Fig. 7 Element importance analysis for hardness prediction in various Cu-based alloys. Each panel displays the analysis for a specific alloy composition as follows: (a) Cu-Cr-Co-Si, (b) Cu-Cr-Ca-Ni-Si, (c) Cu-Cr-Ni-Si, (d) Cu-Cr, (e) Cu-Cr-Zr, (f) Cu-Cr-La-Zr, (g) Cu-Cr-Y-Zr, (h) Cu-Cr-La-Y-Zr, (i) Cu-Ag-Cr-Zn-Zr, (j) Cr-Ag-Cr-Zn, (k) Cu-Cr-Sn-Y-Zn, (l) Cu-Cr-Mg, (m) Cu-Cr-Mg-Si, (n) Cu-Cr-Ce-Sn-Zn, (o) Cu-Cr-Mg-Sn-Zr, (p) Cu-Cr-Mg-Zr, (q) Cu-Cr-Sn-Zr, (r) Cu-Cr-Sn-Zn, (s) Cu-Cr-Yb, (t) Cu-Cr-Co,(u) Cu-Ag-Cr-Zr, (v) Cu-Cr-Ag, (w) Cu-Cr-Sr, (x) Cu-Cr-Ca.

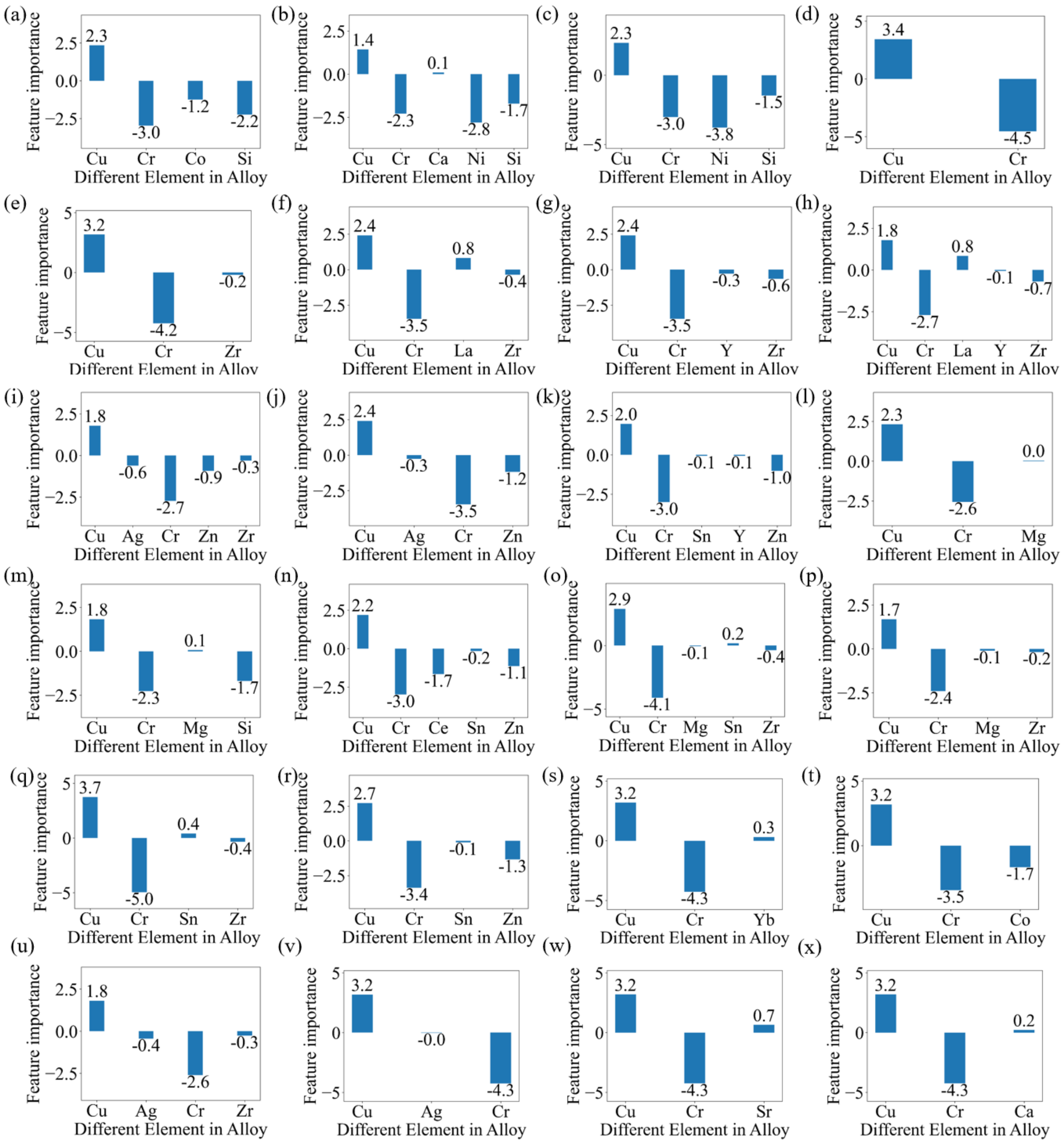

Supplementary Fig. 8 Element importance analysis for conductivity prediction in various Cu-based alloys. Each panel displays the analysis for a specific alloy composition as follows: (a) Cu-Cr-Co-Si, (b) Cu-Cr-Ca-Ni-Si, (c) Cu-Cr-Ni-Si, (d) Cu-Cr, (e) Cu-Cr-Zr, (f) Cu-Cr-La-Zr, (g) Cu-Cr-Y-Zr, (h) Cu-Cr-La-Y-Zr, (i) Cu-Ag-Cr-Zn-Zr, (j) Cr-Ag-Cr-Zn, (k) Cu-Cr-Sn-Y-Zn, (l) Cu-Cr-Mg, (m) Cu-Cr-Mg-Si, (n) Cu-Cr-Ce-Sn-Zn, (o) Cu-Cr-Mg-Sn-Zr, (p) Cu-Cr-Mg-Zr, (q) Cu-Cr-Sn-Zr, (r) Cu-Cr-Sn-Zn, (s) Cu-Cr-Yb, (t) Cu-Cr-Co,(u) Cu-Ag-Cr-Zr, (v) Cu-Cr-Ag, (w) Cu-Cr-Sr, (x) Cu-Cr-Ca.

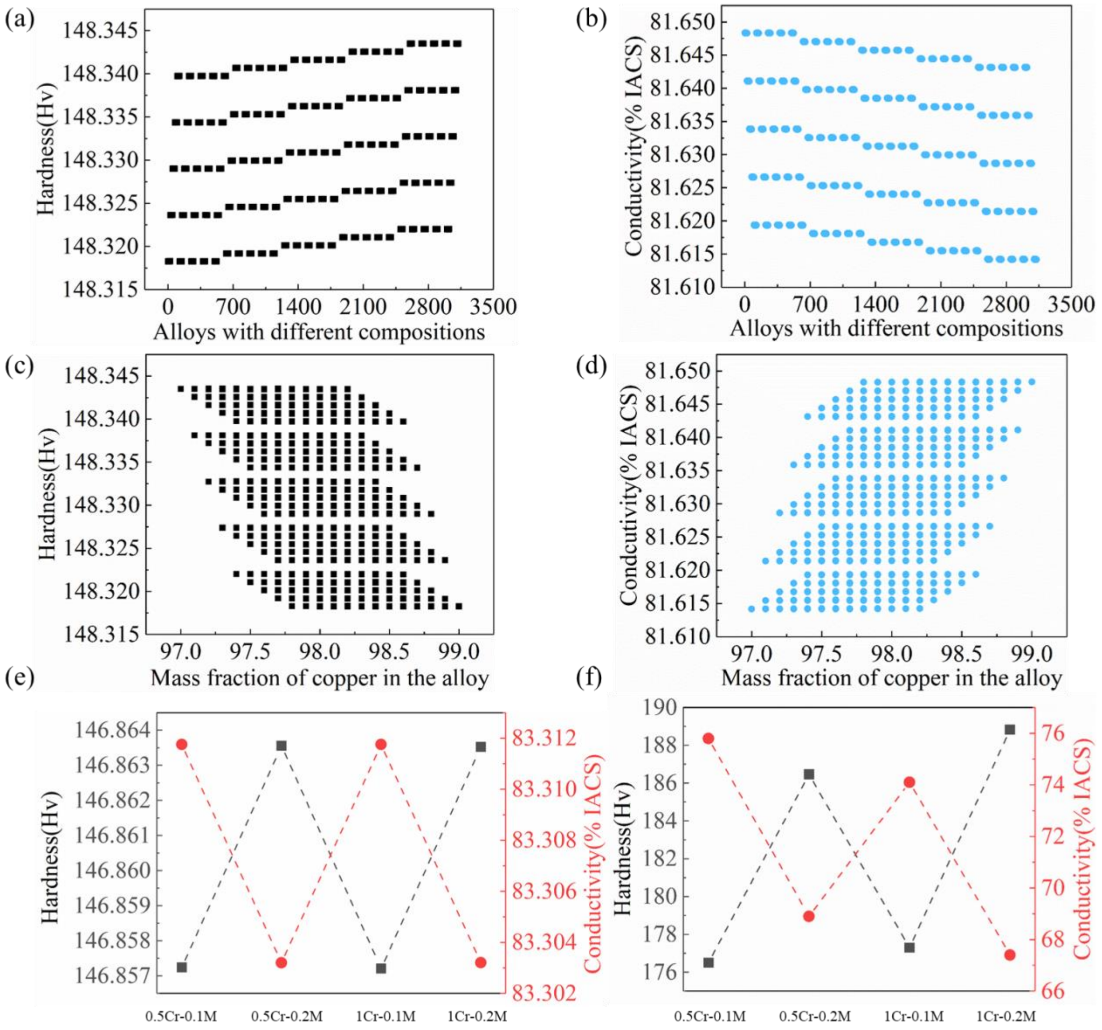

Supplementary Fig. 9 Model Prediction and Experimental Results of Alloy Performance. (a-b) Performance variation results predicted by the model under different alloy compositions. **(a)** Hardness, **(b)** Conductivity. **(c-d)** Performance variation results with the content of copper predicted by the model. **(c)** Hardness, **(d)** Conductivity. **(e)** Performance of Cu-Cr-M Alloys predicted by the model. **(f)** Performance of the Cu-Cr-M Alloy in the Experiment.

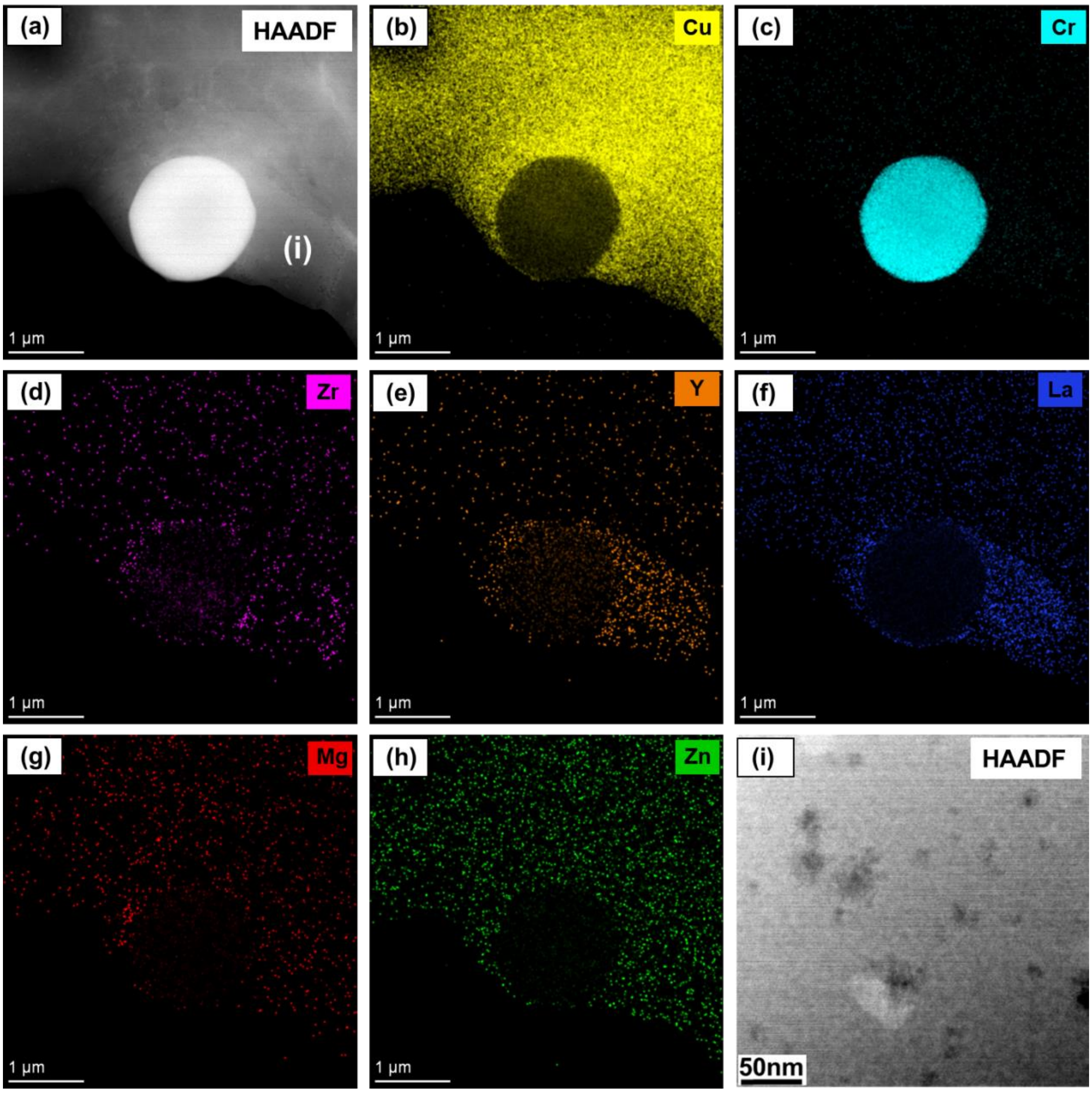

Supplementary Fig. 10 Microstructural characterization of the Cu-Cr-Zr-Y-La-Mg-Zn alloy. (a) Bright-field transmission electron microscopy (TEM) image. **(b-h)** Energy-dispersive X-ray spectroscopy (EDS) elemental maps corresponding to (a). The maps show that Cu, Y, and La enrichment within the nanoprecipitates. **(i)** High-angle annular dark-field (HAADF) image of Cu_5(Y, La) in (a).

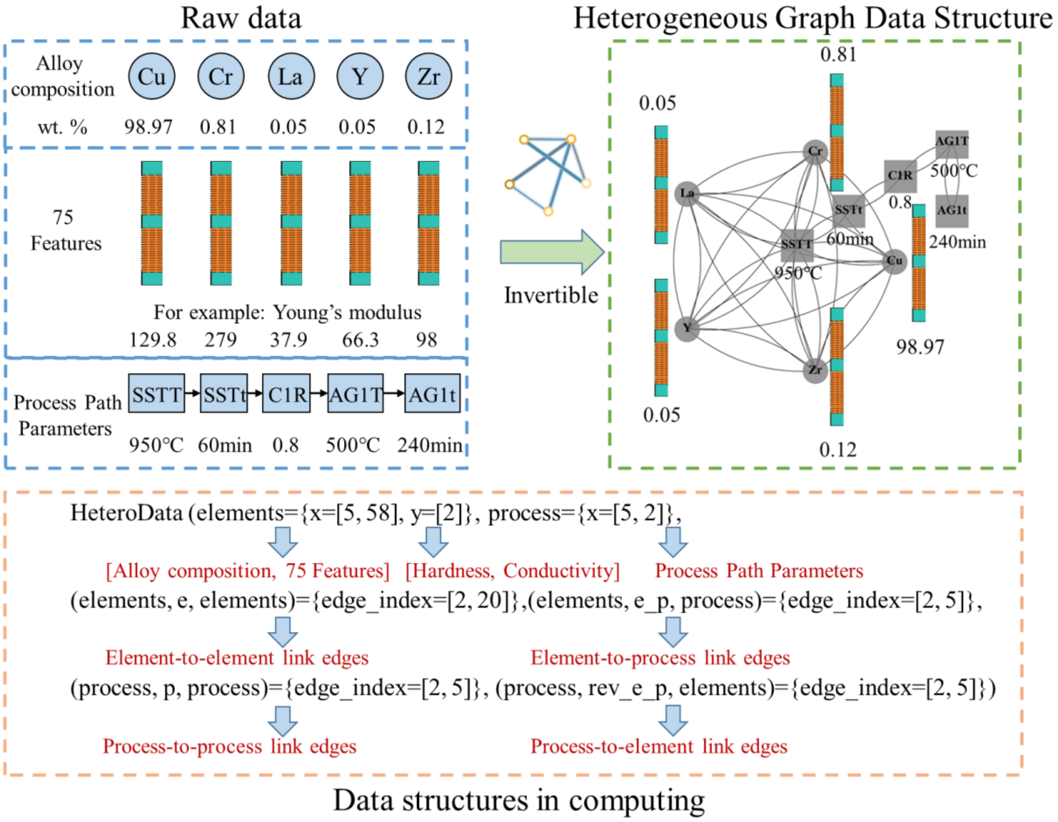

Supplementary Fig. 11 Heterogeneous graph neural network data structure. Raw data, heterogeneous graph data structure, data structure in computing.

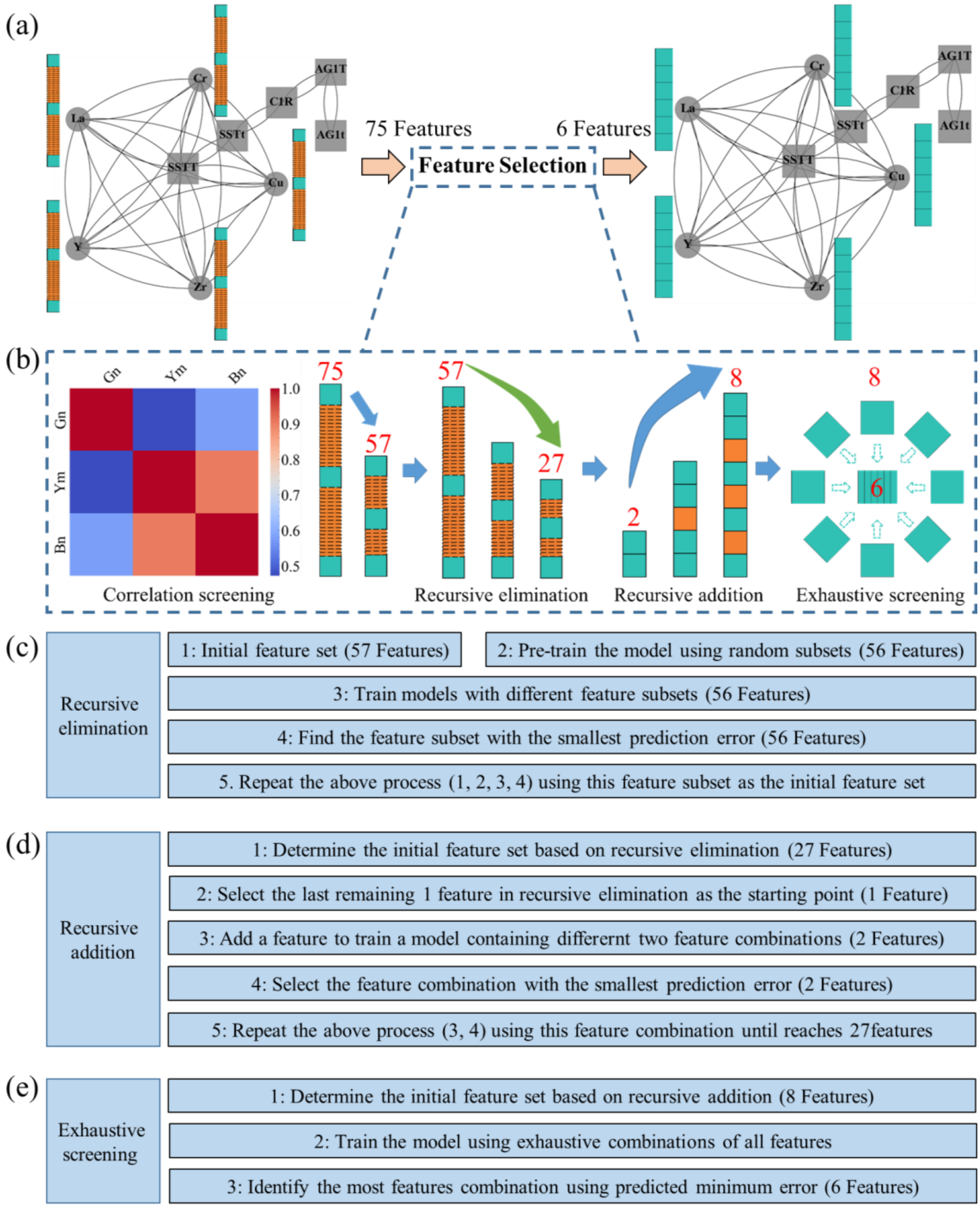

Supplementary Fig. 12 The schematic diagram of the feature screening process. (a-b) Feature screening. The feature selection procedure consists of four sequential steps: correlation analysis, recursive elimination, recursive addition, and exhaustive screening. **(c)** The detailed process of recursive elimination. **(d)** The detailed process of recursive addition. **(e)** The detailed process of exhaustive screening.

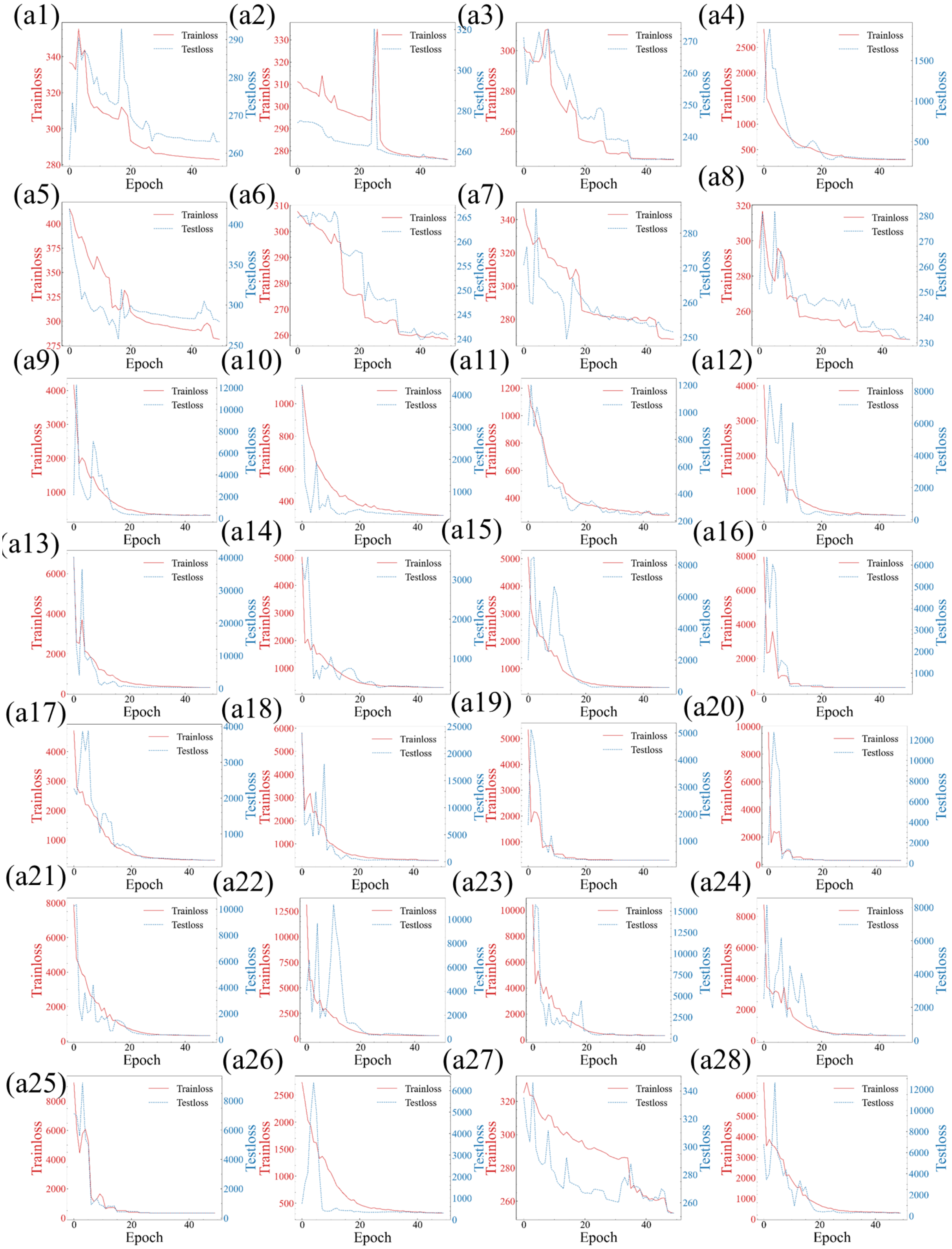

Supplementary Fig. 13 Model pre-training results. Performance of the model during the pre-training phase, evaluated as a function of the number of input features. The numbers indicated on the plot correspond to the quantity of features included in the model for each specific result.

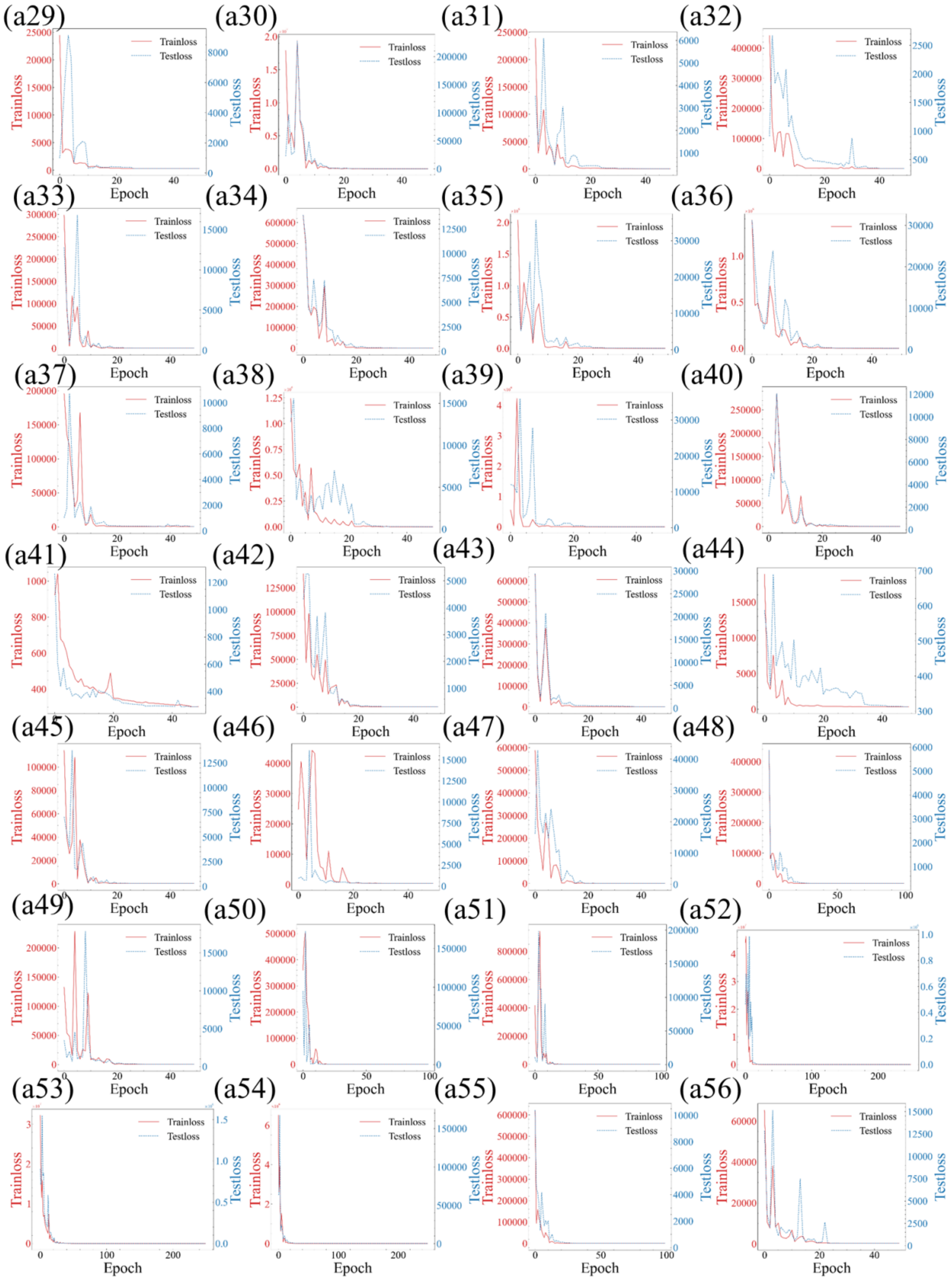

Supplementary Fig. 13 (continued) Model pre-training results. Performance of the model during the pre-training phase, evaluated as a function of the number of input features. The numbers indicated on the plot correspond to the quantity of features included in the model for each specific result.

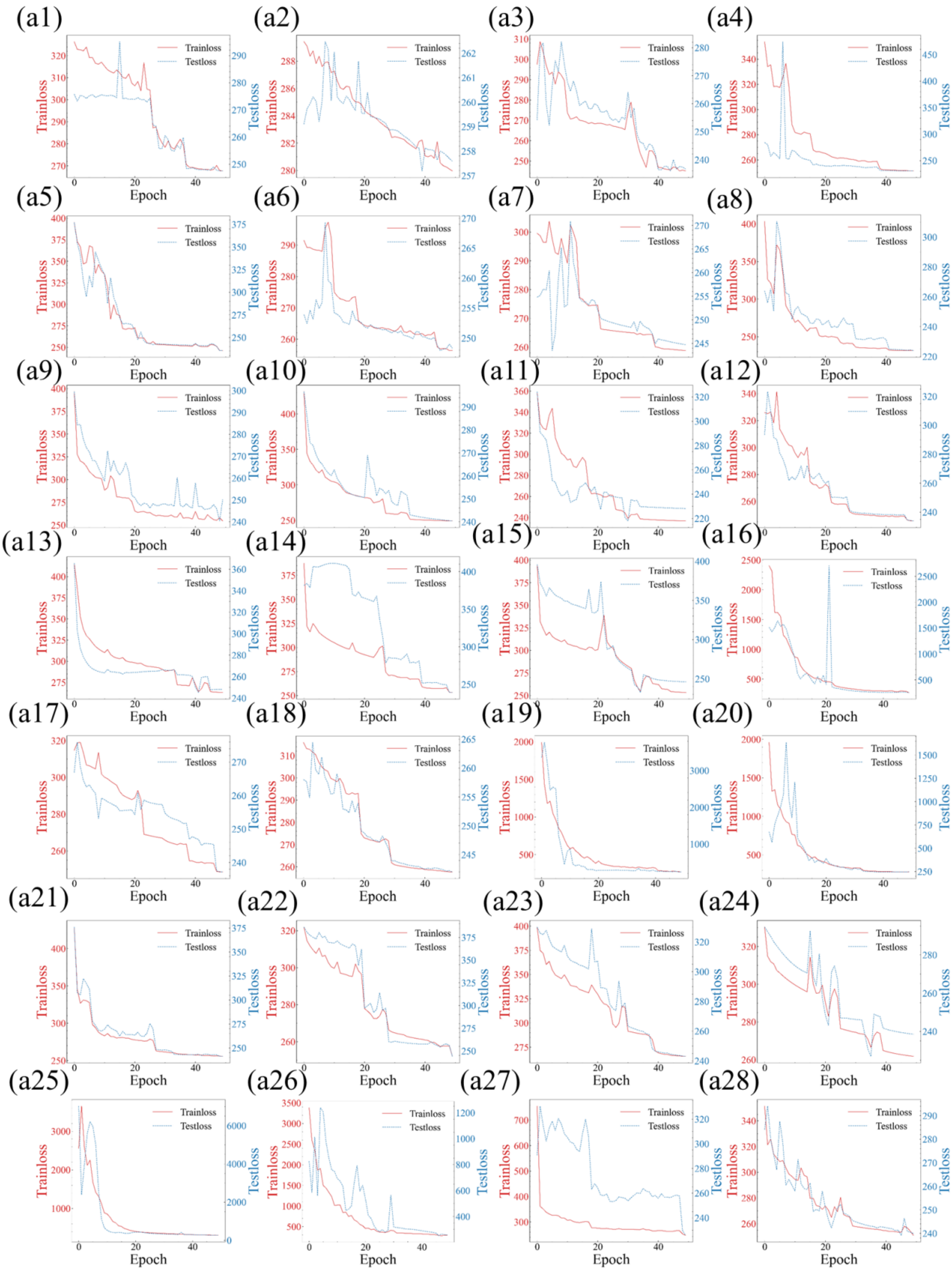

Supplementary Fig. 14 Optimal performance during recursive feature elimination. Each data point represents the minimum training error achieved for a given number of features as they are progressively removed from the model.

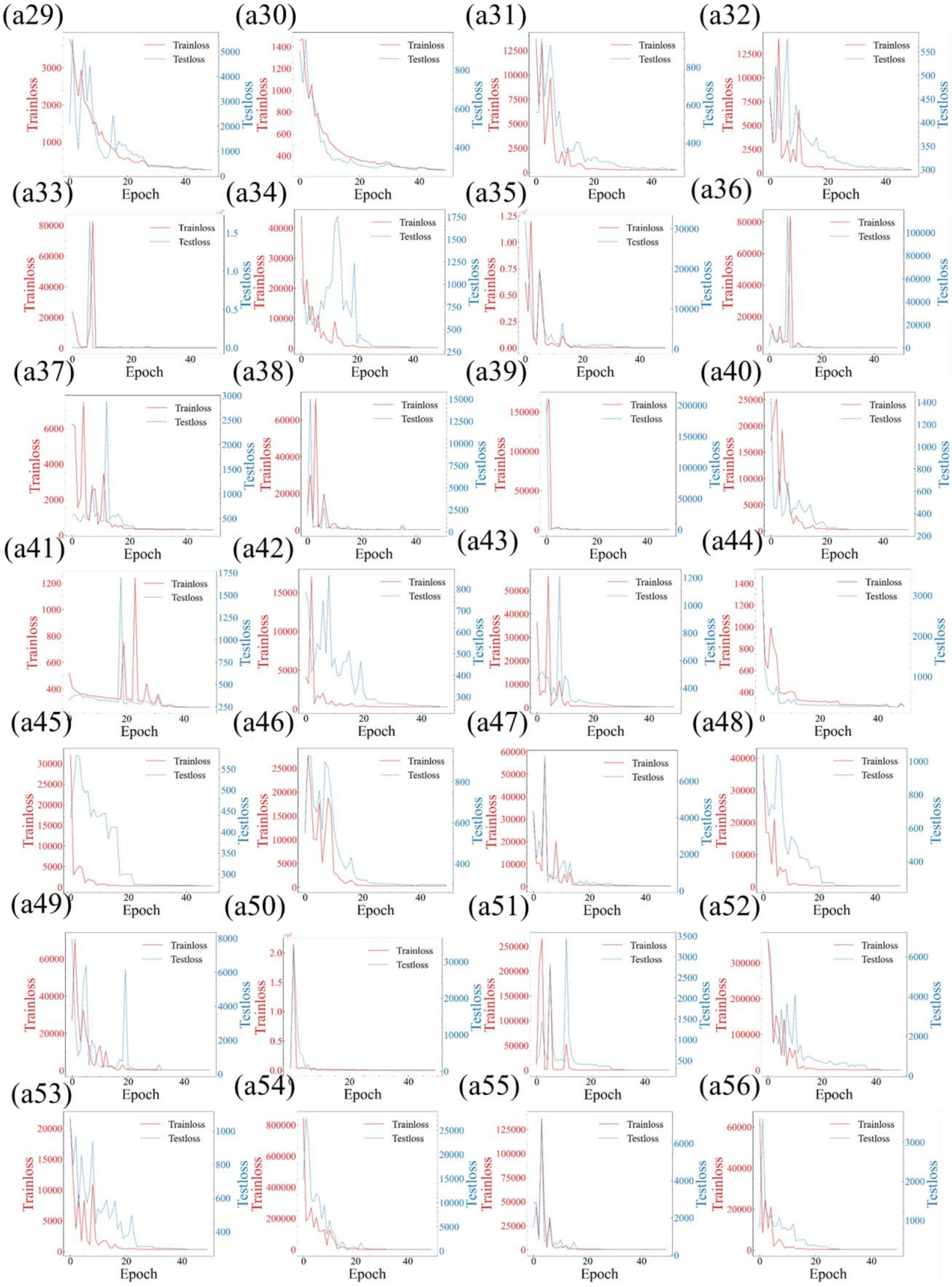

Supplementary Fig. 14 (continued) Optimal performance during recursive feature elimination. Each data point represents the minimum training error achieved for a given number of features as they are progressively removed from the model.

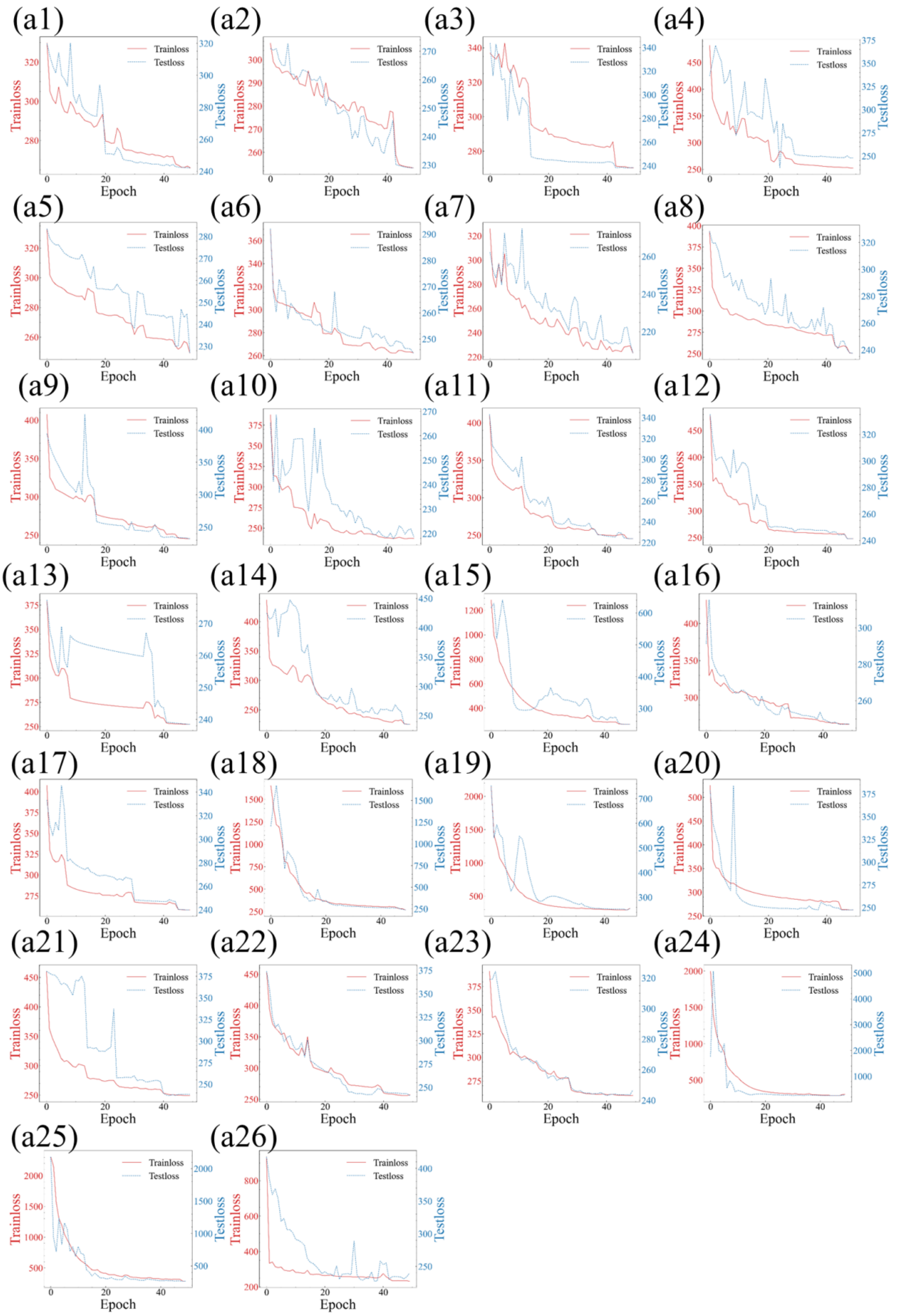

Supplementary Fig. 15 Optimal performance during recursive feature addition. Each data point represents the minimum training error achieved for a given number of features as they are progressively added to the model.

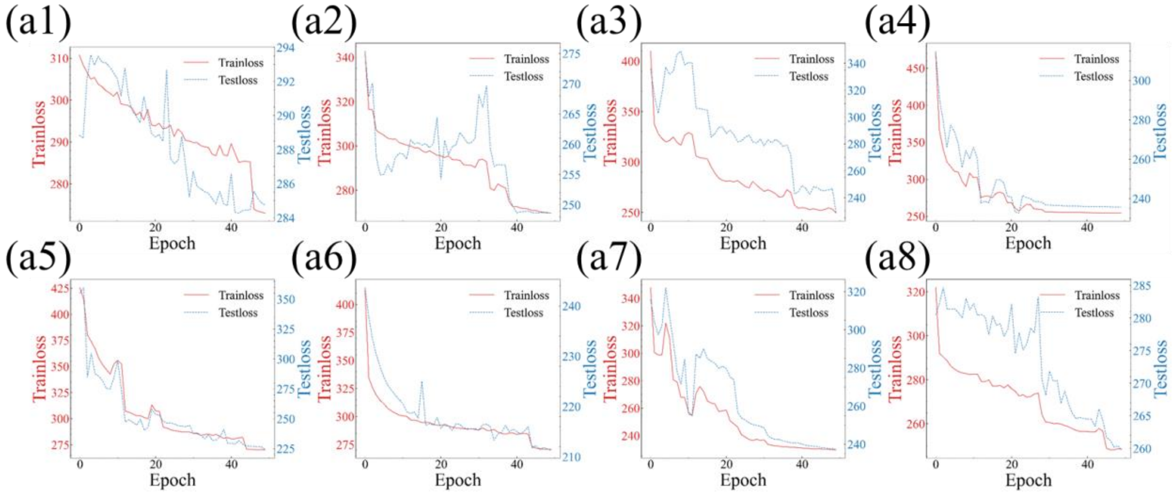

Supplementary Fig. 16 Optimal performance during exhaustive screening. Each data point represents the minimum training error found by testing all possible feature combinations for a given subset size.

Comprehensive Evaluation of Model Performance: Baseline Comparison, Feature Selection, and Robustness Analysis

(1) Comparison with baseline models

To further validate the predictive capability of the proposed model, we trained ten additional commonly used models on the same dataset, including: Bayesian Ridge Regression (BRR), Linear Regression (LR), ElasticNet, Support Vector Regression (SVR), Gradient Boosting Regression (GBR), Random Forest (RF), Decision Tree Regression (DTR), k-Nearest Neighbors (KNN), and Artificial Neural Networks (ANN). The prediction results are shown in Supplementary Fig. 17.

The prediction relative errors of each model on the training, test, and validation sets are shown in Supplementary Fig. 17a-c, respectively. It is important to note that the validation set consists of performance data from our independently fabricated Cu-Cr-Zr-Y-La-Mg-Zn alloys, which were not used during the entire training process. As can be seen from Supplementary Fig. 17a-c, although models such as RF, DTR, and KNN exhibit low prediction errors on the training and test sets, their prediction errors on the validation set are all higher than those of our proposed model. This indicates that our model possesses stronger generalization ability and prediction stability.

Supplementary Fig. 17d-e present a comparison between the predicted values of

different models on the validation set and the experimental values. It can be observed that, for both hardness and electrical conductivity, the prediction points of our proposed model are more concentrated around the ideal line Y=X, demonstrating superior prediction accuracy. Supplementary Fig. 17f-o further compare the ability of each model to capture the trend of validation set samples with the progress of the thermomechanical treatment. The experimental results show that as the thermomechanical treatment proceeds, the alloy hardness increases significantly, while the electrical conductivity decreases slightly. However, Supplementary Fig. 17f-m show that eight models failed to effectively capture the impact of the multi-stage aging treatment: their predictions for the third treatment were almost a single value, falling completely within the prediction range of the second treatment, thus failing to reflect the trend observed experimentally. This exposes the limitations of these models in capturing the evolution patterns of the heat treatment. Supplementary Fig. 17l further indicates that the predictions of the DTR model are completely inconsistent with the experimental results. Furthermore, Supplementary Fig. 17m and n incorrectly predict an increase in electrical conductivity with the progress of the thermomechanical treatment, which contradicts the experimental trend. In contrast, only our proposed model (Supplementary Fig. 17o) can accurately reproduce the consistent trend of hardness and electrical conductivity changes with heat treatment as observed experimentally.

In summary, our model not only achieves the highest accuracy in predicting the properties of new alloys but also demonstrates a high degree of consistency with experimental results in terms of prediction trends, fully proving its superiority and reliability in the task of alloy property prediction.

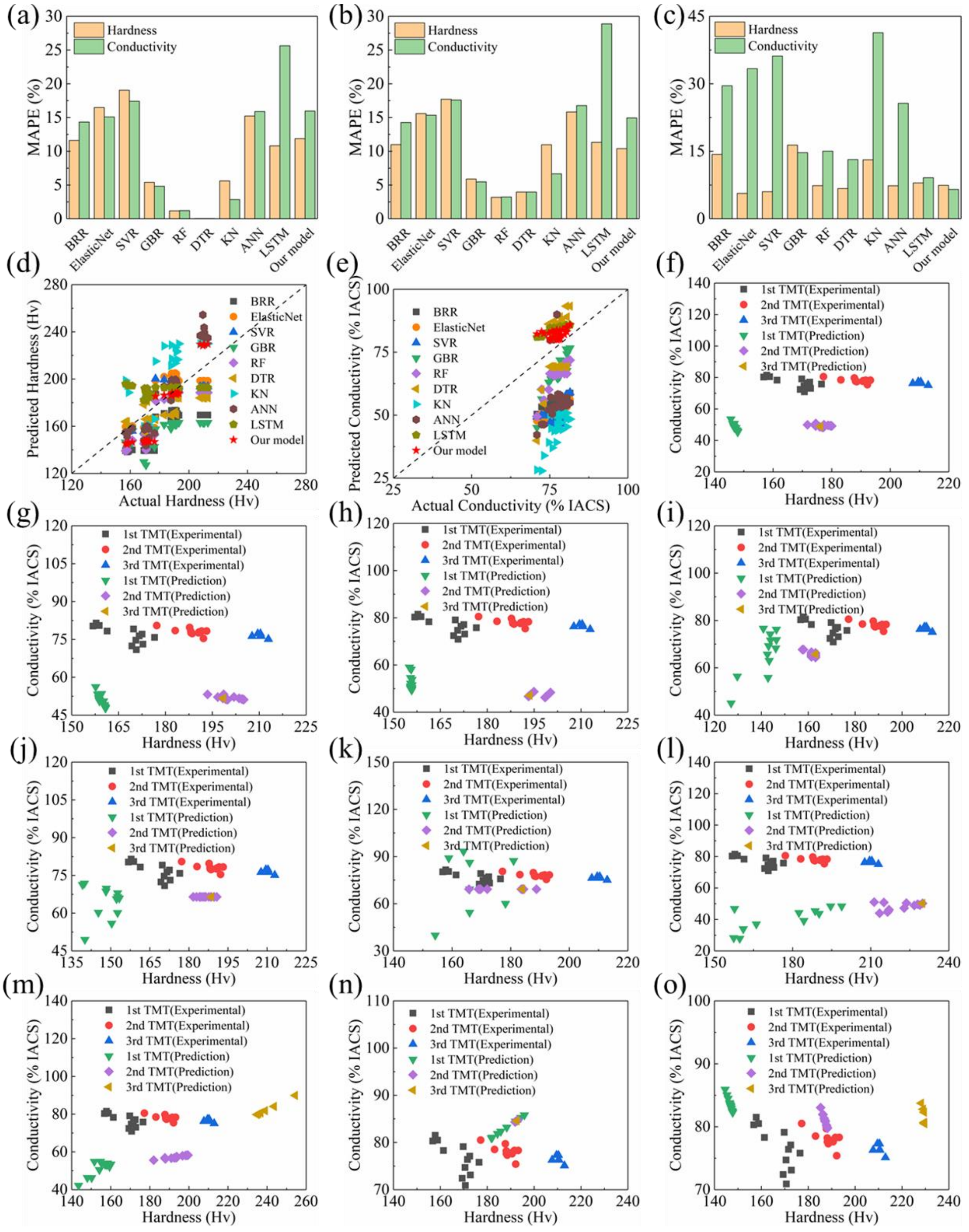

Supplementary Fig. 17 Comparison of Predictive Performance Across Different Models. (a-c) Model Performance Across Diverse Datasets, **(a)** Training set, **(b)** Test set, **(c)** Validation set. **(d-e)** Comparison of model predictions on the validation set, **(d)** Hardness, **(e)** Conductivity. **(f-o)** Comparing model-predicted and experimentally measured results on the validation set, **(f)** Bayesian Ridge Regression (BRR), **(g)** ElasticNet, **(h)** Support Vector Regression (SVR), **(i)** Gradient Boosting Regression (GBR), **(j)** Random Forest (RF), **(k)** Decision Tree Regression (DTR), **(l)** k-Nearest

Neighbors (KNN), **(m)** Artificial Neural Networks (ANN), **(n)** Long Short-Term Memory (LSTM), and **(o)** Our model.

(2) Effectiveness of the feature selection strategy

To validate the effectiveness of the feature selection method adopted in this study, we systematically compared three different feature selection strategies: the proposed method, the approach based on SHAP analysis, and the method based on the genetic algorithm. Specifically, we employed both the SHAP method and a genetic algorithm to screen the original feature set, and subsequently rebuilt the models using the selected features. After completing model training, we identified the optimal parameters for performance prediction and systematically compared the predictive performance of the models under the three feature selection strategies. The results are summarized in Supplementary Fig. 18.

As shown in Supplementary Fig. 18a-c, we systematically compared the predictive performance of the three feature selection strategies. Our method (Supplementary Fig. 18a) demonstrated superior generalization capability. In contrast, the model based on SHAP-selected features (Supplementary Fig. 18b), while achieving prediction errors on the training and test sets comparable to our method, exhibited a significantly higher error on the validation set. Furthermore, the model constructed using features selected by the genetic algorithm (Supplementary Fig. 18c) showed higher prediction errors across the training, test, and validation sets. These results indicate that the feature selection strategy employed in our study possesses a distinct advantage in enhancing model generalization capability.

Further comparing the prediction trends of each model on the validation set (Supplementary Fig. 18d-f), it can be observed that the performance trends predicted by our method are highly consistent with the experimental results. In contrast, the prediction trend of the model based on SHAP-selected features deviated noticeably from the experimental outcome: as the thermomechanical treatment process proceeded, the experimental results showed a gradual decrease in electrical conductivity, whereas the SHAP-based predictions exhibited an increasing trend. For the features selected by the genetic algorithm, although the predicted trend aligned with the experiment, its

prediction accuracy was still inferior to that of our proposed method.

Furthermore, we conducted an in-depth examination of the SHAP analysis results. The findings revealed that among all 57 initial features, the three core features selected in our study were all ranked within the top 20 in terms of SHAP importance (as highlighted by the circle in Supplementary Fig. 18g). This discovery not only demonstrates that the selected features are highly consistent with the important features identified by the SHAP method, but also provides strong support for their validity from a statistical learning perspective. It further validates the scientific soundness and effectiveness of the feature selection strategy adopted in this research.

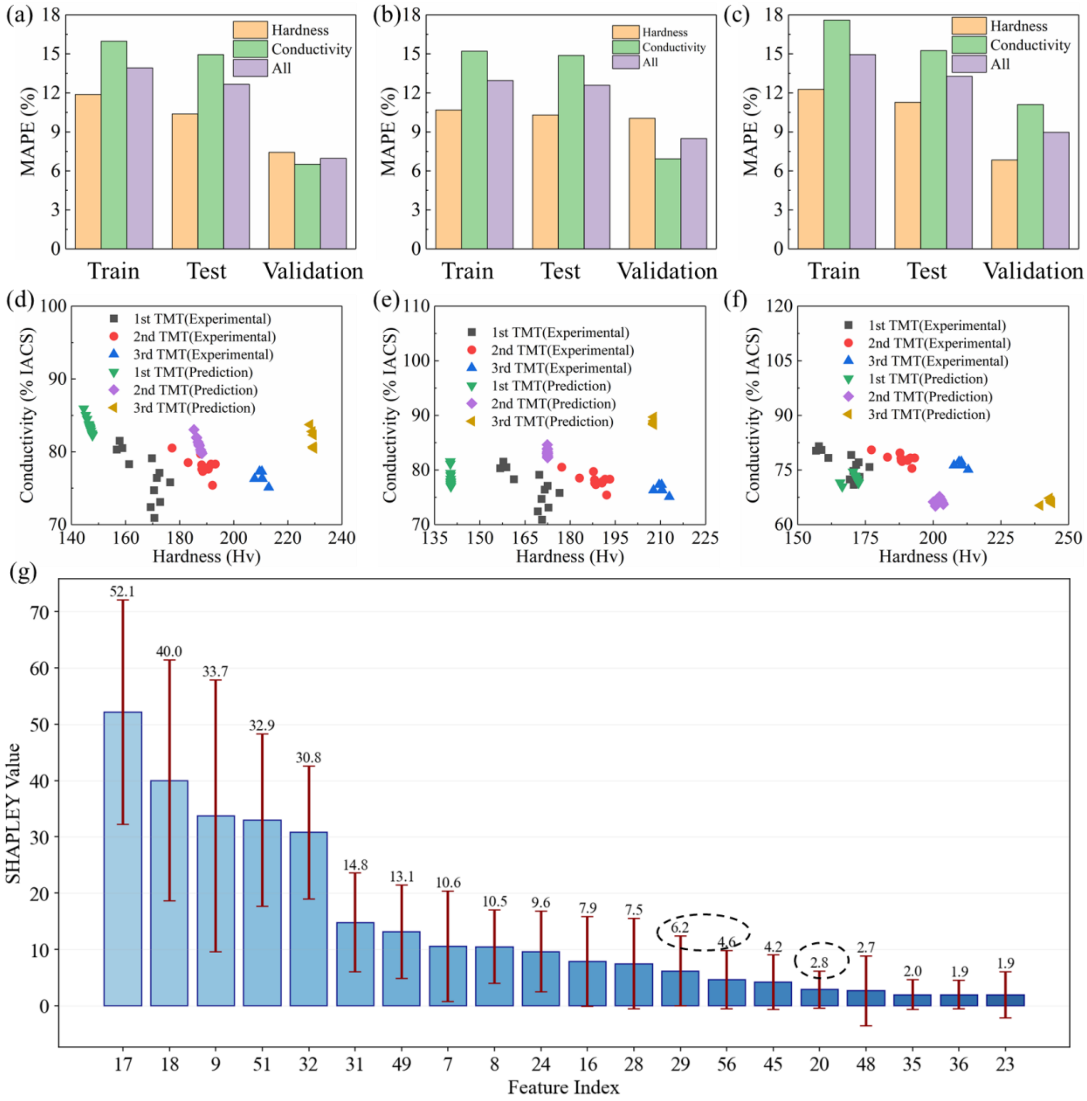

Supplementary Fig. 18 Predictive performance of models using different feature selection methods. (a-c) Predictive performance of models on different datasets using

various feature selection methods, **(a)** Our method, **(b)** Based on the SHAP analysis, and **(c)** Based on the genetic algorithm. **(d-f)** Comparison of predicted and experimental results on different datasets using various feature selection methods, **(d)** Our method, **(e)** Based on the SHAP analysis, and **(f)** Based on the genetic algorithm. **(g)** The 20 most important features were selected from the 57 based on SHAP analysis. The circled areas indicate the key features selected by our method.

(3) Robustness of the selected features under different random seeds

To assess the robustness of the feature importance rankings, we retrained the model using four different random seeds, with the results shown in the Supplementary Fig. 19-20. Despite slight numerical variations in the influence of key physical descriptors across different initializations, their relative rankings remain consistent. This consistency confirms that the model indeed captures intrinsic material characteristics rather than statistical biases.

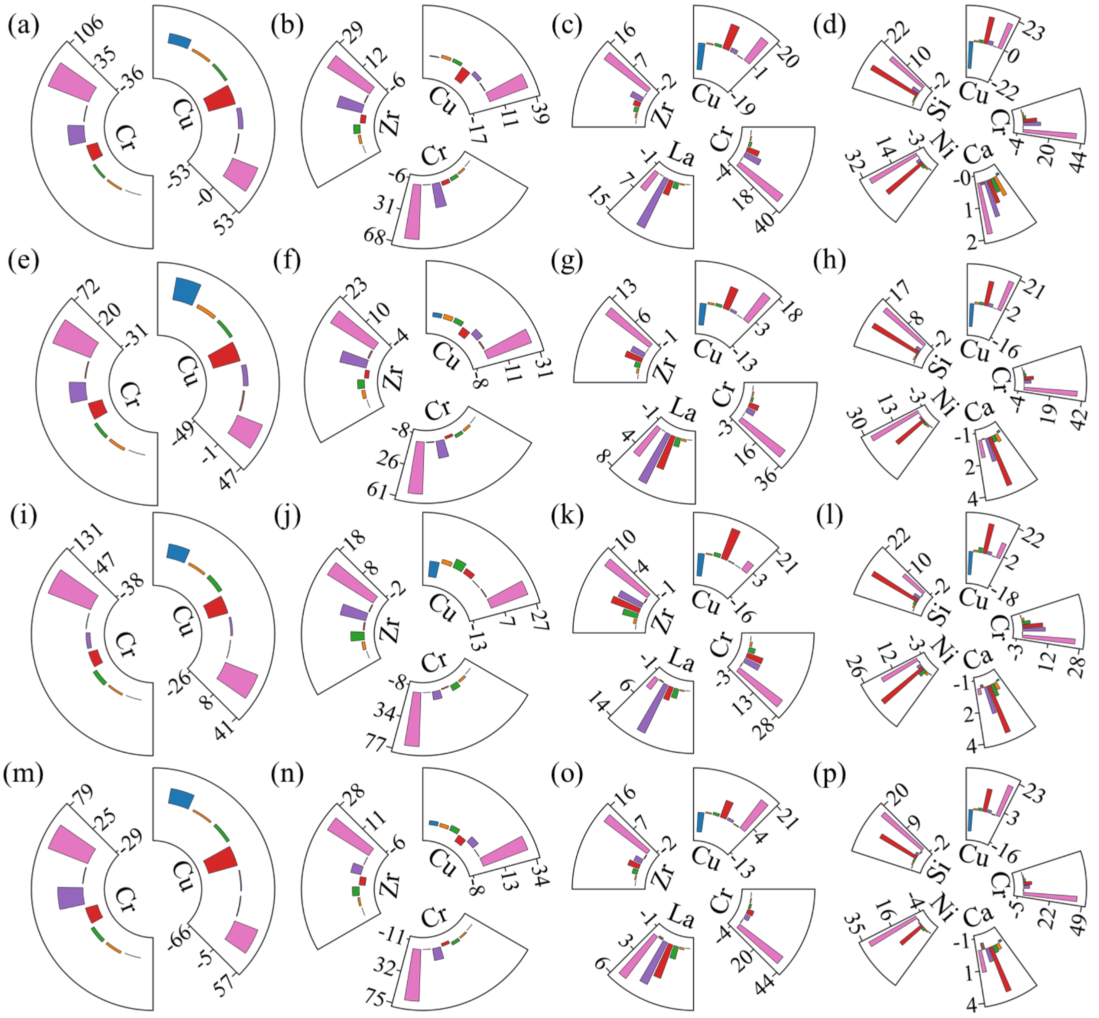

Supplementary Fig. 19 Comparative identification of key features affecting alloy hardness under varying random seed configurations. (a-d) Random seed = 42: **(a)** Cu-Cr (binary alloy), **(b)** Cu-Cr-Zr (ternary alloy), **(c)** Cu-Cr-La-Zr (quaternary alloy), and **(d)** Cu-Cr-Ca-Ni-Si (quinary alloy). **(e-h)** Random seed = 1314: **(e)** Cu-Cr (binary alloy), **(f)** Cu-Cr-Zr (ternary alloy), **(g)** Cu-Cr-La-Zr (quaternary alloy), and **(h)** Cu-Cr-Ca-Ni-Si (quinary alloy). **(i-l)** Random seed = 888: **(i)** Cu-Cr (binary alloy), **(j)** Cu-Cr-Zr (ternary alloy), **(k)** Cu-Cr-La-Zr (quaternary alloy), and **(l)** Cu-Cr-Ca-Ni-Si (quinary alloy). **(m-p)** Random seed = 10001: **(m)** Cu-Cr (binary alloy), **(n)** Cu-Cr-Zr (ternary alloy), **(o)** Cu-Cr-La-Zr (quaternary alloy), and **(p)** Cu-Cr-Ca-Ni-Si (quinary alloy).

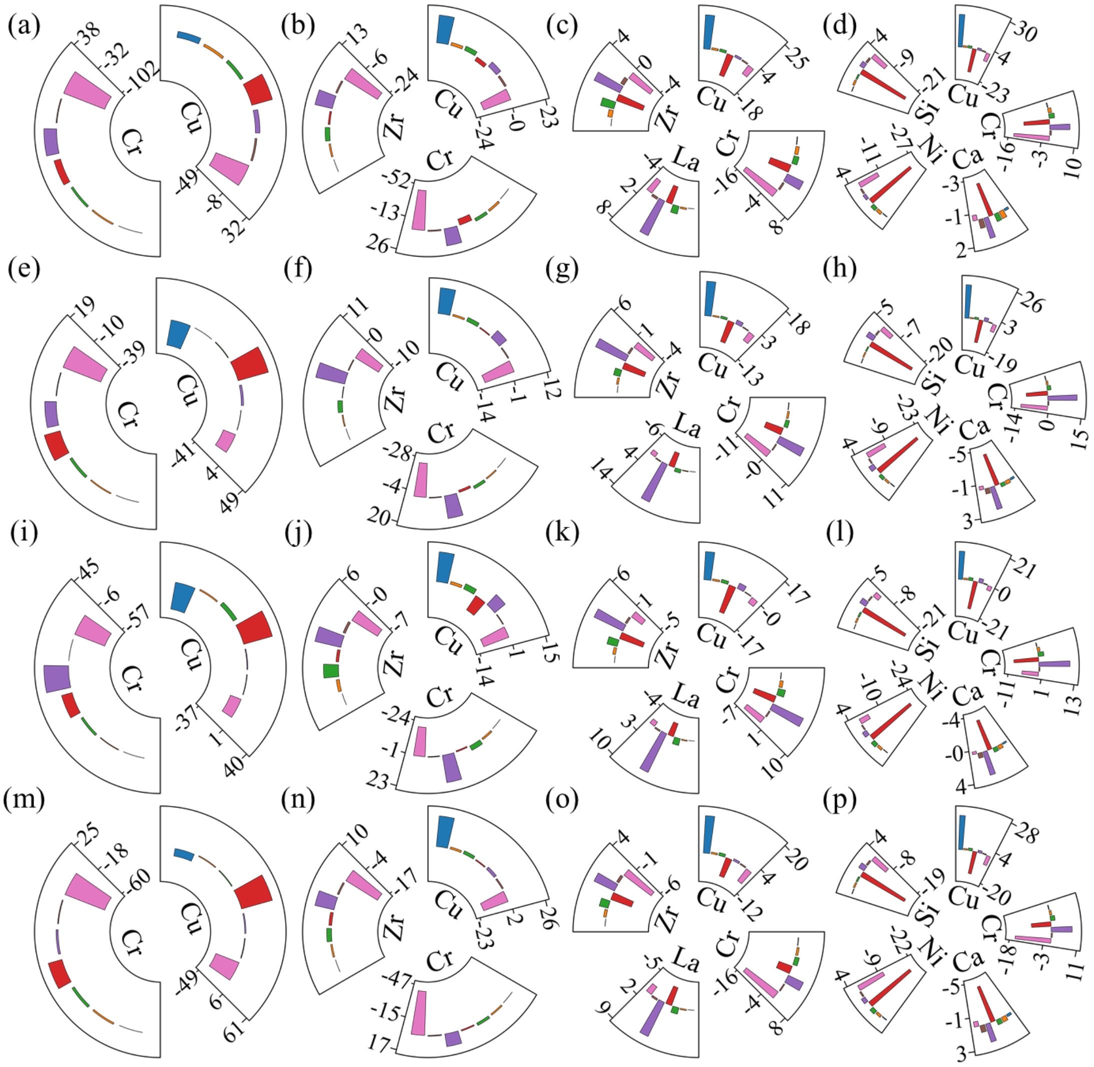

Supplementary Fig. 20 Comparative identification of key features affecting alloy conductivity under varying random seed configurations. (a-d) Random seed = 42:

(a) Cu-Cr (binary alloy), **(b)** Cu-Cr-Zr (ternary alloy), **(c)** Cu-Cr-La-Zr (quaternary alloy), and **(d)** Cu-Cr-Ca-Ni-Si (quinary alloy). **(e-h)** Random seed = 1314: **(e)** Cu-Cr (binary alloy), **(f)** Cu-Cr-Zr (ternary alloy), **(g)** Cu-Cr-La-Zr (quaternary alloy), and **(h)** Cu-Cr-Ca-Ni-Si (quinary alloy). **(i-l)** Random seed = 888: **(i)** Cu-Cr (binary alloy), **(j)** Cu-Cr-Zr (ternary alloy), **(k)** Cu-Cr-La-Zr (quaternary alloy), and **(l)** Cu-Cr-Ca-Ni-Si (quinary alloy). **(m-p)** Random seed = 10001: **(m)** Cu-Cr (binary alloy), **(n)** Cu-Cr-Zr (ternary alloy), **(o)** Cu-Cr-La-Zr (quaternary alloy), and **(p)** Cu-Cr-Ca-Ni-Si (quinary alloy).
